# Ecological niche modeling of *Aedes* mosquito vectors of chikungunya virus in southeastern Senegal

**DOI:** 10.1186/s13071-018-2832-6

**Published:** 2018-04-19

**Authors:** Rebecca Richman, Diawo Diallo, Mawlouth Diallo, Amadou A. Sall, Oumar Faye, Cheikh T. Diagne, Ibrahima Dia, Scott C. Weaver, Kathryn A. Hanley, Michaela Buenemann

**Affiliations:** 1 Department of Geography, New Mexico State University, Las Cruces, NM USA; 2 Department of Biology, New Mexico State University, Las Cruces, NM USA; 3 Institut Pasteur de Dakar, Dakar, Senegal; 4 Laboratoire d’Écologie Vectorielle et Parasitaire, Université Cheikh Anta Diop, Dakar, Senegal; 5 Institute for Human Infections and Immunity, Center for Tropical Diseases, and Department of Microbiology and Immunology, University of Texas Medical Branch, Galveston, Texas USA

**Keywords:** Chikungunya virus, Maxent, Arbovirus, Ecological niche model, Mosquitoes, *Aedes*, Environmental factors, Senegal

## Abstract

**Background:**

Chikungunya virus (CHIKV) originated in a sylvatic cycle of transmission between non-human animal hosts and vector mosquitoes in the forests of Africa. Subsequently the virus jumped out of this ancestral cycle into a human-endemic transmission cycle vectored by anthropophilic mosquitoes. Sylvatic CHIKV cycles persist in Africa and continue to spill over into humans, creating the potential for new CHIKV strains to enter human-endemic transmission. To mitigate such spillover, it is first necessary to delineate the distributions of the sylvatic mosquito vectors of CHIKV, to identify the environmental factors that shape these distributions, and to determine the association of mosquito presence with key drivers of virus spillover, including mosquito and CHIKV abundance. We therefore modeled the distribution of seven CHIKV mosquito vectors over two sequential rainy seasons in Kédougou, Senegal using Maxent.

**Methods:**

Mosquito data were collected in fifty sites distributed in five land cover classes across the study area. Environmental data representing land cover, topographic, and climatic factors were included in the models. Models were compared and evaluated using area under the receiver operating characteristic curve (AUROC) statistics. The correlation of model outputs with abundance of individual mosquito species as well as CHIKV-positive mosquito pools was tested.

**Results:**

Fourteen models were produced and evaluated; the environmental variables most strongly associated with mosquito distributions were distance to large patches of forest, landscape patch size, rainfall, and the normalized difference vegetation index (NDVI). Seven models were positively correlated with mosquito abundance and one (*Aedes taylori*) was consistently, positively correlated with CHIKV-positive mosquito pools. Eight models predicted high relative occurrence rates of mosquitoes near the villages of Tenkoto and Ngary, the areas with the highest frequency of CHIKV-positive mosquito pools.

**Conclusions:**

Of the environmental factors considered here, landscape fragmentation and configuration had the strongest influence on mosquito distributions. Of the mosquito species modeled, the distribution of *Ae. taylori* correlated most strongly with abundance of CHIKV, suggesting that presence of this species will be a useful predictor of sylvatic CHIKV presence.

**Electronic supplementary material:**

The online version of this article (10.1186/s13071-018-2832-6) contains supplementary material, which is available to authorized users.

## Background

Mosquito-borne chikungunya virus (CHIKV) was first identified in Tanzania in 1953. Until 2004, CHIKV was considered a minor tropical pathogen responsible for a small number of cases of chikungunya fever, typified by high fevers and severe joint pain but not death [1]. However, in 2005 and 2006 chikungunya disease incidence surged, with more than 272,000 CHIKV cases and up to 225 deaths in the Indian Ocean islands as well as 1.5 million cases in India [1, 2]. In 2007, chikungunya fever cases were reported in Europe for the first time [1, 3]. Most recently, CHIKV established autochthonous transmission in the Americas; the first cases were reported from the Caribbean island of Saint Martin in December 2013 and the virus has since spread across South America, Central America, and into the USA [4–9].

CHIKV originated in Africa in a sylvatic cycle in which non-human primates and possibly other animals, such as rodents, squirrels and cattle, serve as reservoir hosts [10]. Sylvatic CHIKV exists as two genetically-distinct lineages: one in West Africa (the West African lineage) and the other in East, Central, and South Africa (the ECSA lineage) [9]. The latter has jumped into a human-endemic cycle, which was initially transmitted predominantly by the domestic mosquito *Aedes aegypti* [9]. Phylogenetic evidence indicates that the human-endemic cycle is comprised of an Asian and an Indian Ocean lineage, each of which arose independent from the ESCA lineage [9, 11]. Additionally, a series of envelope glycoprotein gene mutations in the Indian Ocean lineage allowed this virus to be transmitted by the peridomestic mosquito species *Ae. albopictus*. *Aedes albopictus* is more cold-hardy than *Ae. aegypti* and consequently this vector switch enabled transmission of CHIKV in more temperate areas [12, 13].

Thus, sylvatic CHIKV has a well-documented history of emerging into human-endemic transmission and launching widespread epidemics. Moreover, sylvatic CHIKV continues to spill over into humans living near foci of sylvatic transmission in Africa and to cause disease there [10, 14]. In particular, our research team has demonstrated the circulation and spillover of sylvatic CHIKV in the Department of Kédougou in southeastern Senegal, where several other sylvatic arthropod-borne viruses (arboviruses) including dengue, yellow fever and Zika virus, also occur [14–26]. Kédougou is the site of fifty years of continuous mosquito and arbovirus surveillance by the Institut Pasteur de Dakar, Senegal. Moreover we recently concluded an intensive five-year study (2009–2013) of the spatio-temporal distribution and abundance of arboviruses and their mosquito vectors in Kédougou. Over the course of these studies CHIKV has been detected in multiple mosquito species, primarily *Ae. africanus*, *Ae. aegypti formosus*, *Ae. dalzieli*, *Ae. furcifer*, *Ae. luteocephalus*, *Ae. taylori* and *Ae. vittatus* [17, 18, 22, 23]. CHIKV is amplified (i.e. is detected during regular screening of homogenized groups or “pools” of mosquitoes) cyclically in these vectors at roughly four-year intervals; evidence of virus infection is often also detected in monkeys [African green monkeys (*Chlorocebus sabaeus*), patas monkeys (*Erythrocebus patas*), and Guinea baboons (*Papio papio*)] and humans during amplifications [14, 16, 18, 19]. Our intensive ecological study encompassed a CHIKV amplification in 2009 [23].

To mitigate sylvatic CHIKV spillover and thereby diminish the likelihood of emergence of new human-endemic lineages, it is first necessary to delineate the distributions of sylvatic mosquito vectors of CHIKV, to identify the environmental factors that shape these distributions, and to determine associations between the occurrence of particular mosquito species with the abundance of that species and also the abundance of CHIKV, e.g. [27–33]. In our study of the ecology of sylvatic CHIKV circulation in Kédougou [23], we first tested whether broad classifications of land cover could explain the distribution of CHIKV and its vectors. We identified five major classes of land cover (village, savanna, agriculture, barren and forest) in the region and collected mosquitoes at replicate sites within each. While we found complex associations between the abundance of particular CHIKV vector species and land cover type, most species were found in all land covers sampled. To our surprise, we did not find significant differences among the land cover classes in the frequency of mosquito pools positive for CHIKV, although we did detect significant spatial variation in the distribution of CHIKV-positive pools. We concluded that more fine-grained analyses of the associations of environmental factors with mosquito distributions and CHIKV distributions were needed in order to predict the distribution of viruses and vectors and to identify the factors that shape these distributions. Thus, in the current study we undertook an ecological niche analysis of the seven putative CHIKV vectors listed above. This study utilized data from the rainy seasons of 2009 and 2010. We used data from 2009 because that was the year during which a CHIKV amplification occurred; we used data from 2010 to assess the temporal stability of mosquito distributions. Because mosquito abundance may be a stronger predictor of disease rates than mosquito presence, we tested whether model predictions of presence of a particular species correlated with our measures of abundance of that species. The ultimate goal of our research program is to enhance prediction and prevention of CHIKV spillover, so we also tested whether model predictions of a particular species’ presence correlated with abundance of CHIKV-positive mosquito pools.

## Methods

### Study area

**Fig. 1:**
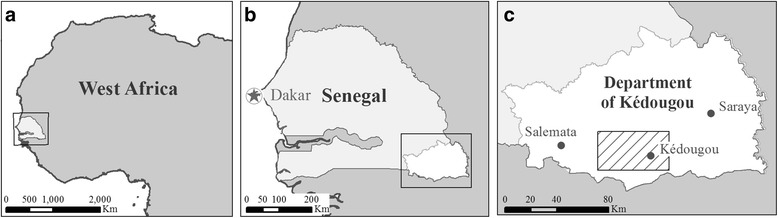
Location of the study area in the Kédougou Department (**c**) of southeastern Senegal (**b**) in western Africa (**a**)

**Fig. 2:**
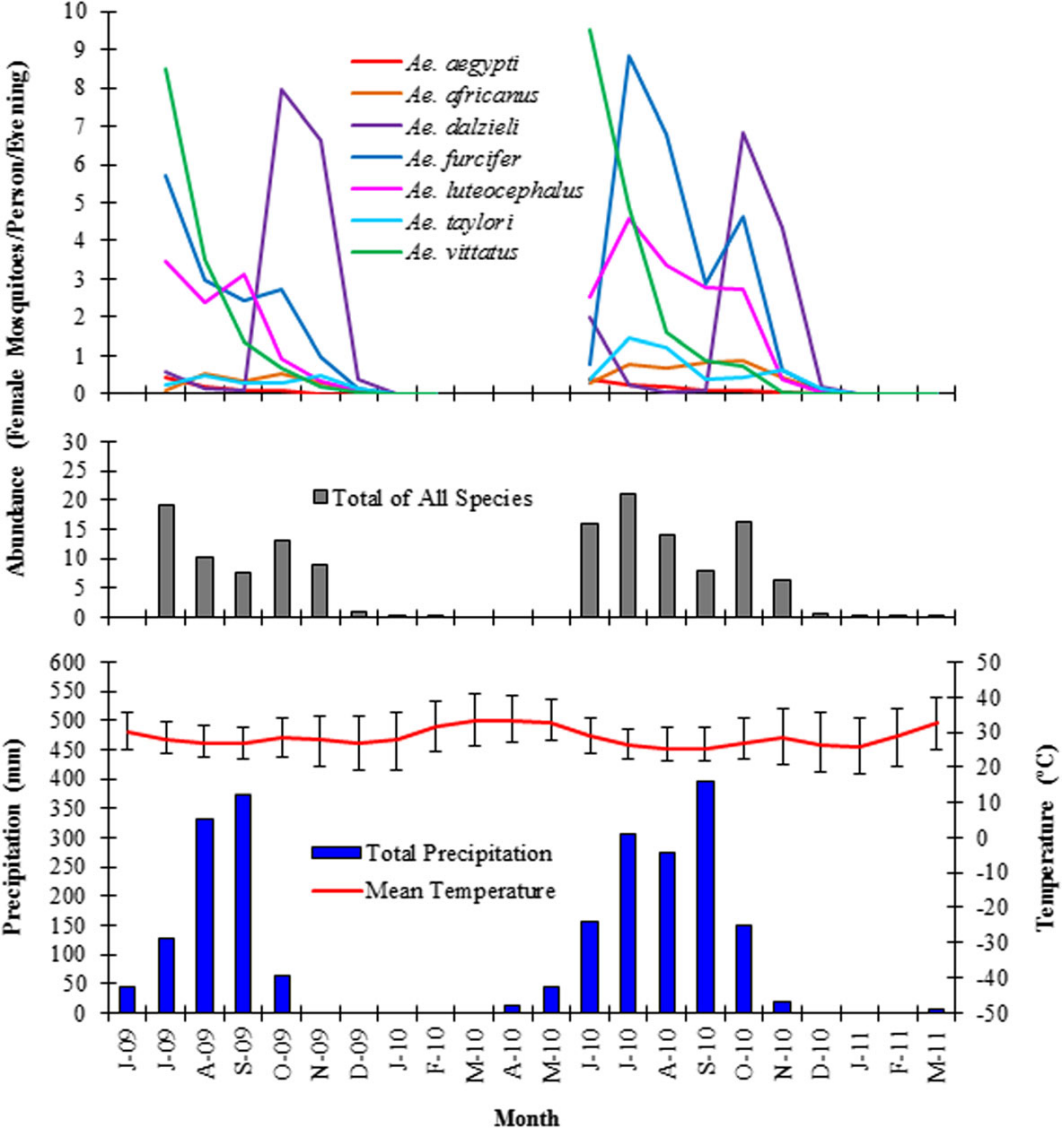
Mosquito abundance, precipitation, and temperature for June 2009 - March 2011. The 2009 data were previously presented in [23]. The June 2009 abundance data are not shown here because only one study block was sampled during that month. Precipitation and temperature data are for Kédougou, Senegal (12°34'1.2"N, 12°13'1.2"W; 178 m a.s.l; [100]). Error bars on the temperature line indicate mean maximum and minimum monthly temperatures. Precipitation and temperature vary slightly across the study area, but trends displayed in the graph area are representative of the entire study area

The study area encompassed 1650 km^2^ in the Kédougou Department in southeastern Senegal (Fig. 1). The area is characterized by a tropical savanna climate [34] with one dry (generally December to May) and one wet (generally June to November) season (Fig. 2). The Kédougou region has traditionally been sparsely populated (mean of 4 people/km^2^) but recent expansion of gold mining in the area has increased the population with migrants coming from Mali, Guinea, Gambia, Ghana, Burkina Faso, Togo and Nigeria [35, 36]. The study area is mostly rural with only one urban center, the town of Kédougou. The landscape can be divided into five major land cover classes: savanna (74.3% of the study area), agricultural land (7.9%), forest (12.5%), barren land (5.0%) and villages (0.1%) ([23]; percentages are representative of 11 June 2009).

There is high mosquito diversity in Kédougou: 102 species have been collected using a combination of human landing collections, light traps, and animal baited traps [18]. Fifty mosquito species from six genera were collected *via* human land collections as part of our study [23], of which seven (i.e. *Ae. africanus*, *Ae. aegypti formosus*, *Ae. dalzieli*, *Ae. furcifer*, *Ae. luteocephalus*, *Ae. taylori* and *Ae. vittatus*) are known to be competent vectors of sylvatic CHIKV in laboratory studies and/or to show high rates of infection in the field [17–19, 21, 23]. These seven species are classified as tree-hole mosquitoes, although Diallo et al. [22] found larvae of all but one (*Ae. africanus*) in other water-holding containers, including fresh fruit husks, decaying fruit husks, puddles, bamboo holes, discarded containers, tires, rocks holes and storage containers.

### Overview of research data and methods

**Fig. 3:**
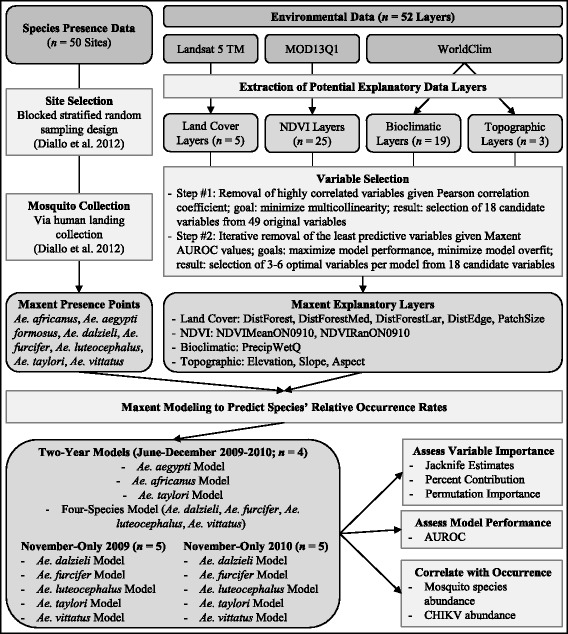
Methods flowchart. See Methods section for details. *Abbreviations*: DistForest, distance from any forest patch; DistForestMed, distance from medium to large forest patches; DistForestLar, distance from large forest patches; DistEdge, distance from patch edge; PatchSize, size of patch; NDVIMeanON0910, mean NDVI for 2009 and 2010 October-November; NDVIRanON0910, range of NDVI for 2009 and 2010 rainy seasons; PrecipWetQ, precipitation of the wettest quarter; CHIKV, chikungunya virus

The workflow for data collection and analysis is summarized in Fig. 3. Briefly, we integrated detection of mosquito occurrences with data on environmental factors thought to influence mosquito species distributions in a series of fourteen Maxent models. Fifty sites for mosquito collection were selected using a blocked stratified random sampling design and mosquitoes in these sites were then collected using human landing collections. Fifty-two environmental data layers representing land cover, Normalized Difference Vegetation Index (NDVI), bioclimatic and topographic variables were derived from three sources (Landsat 5 TM, MOD13Q1, WorldClim). Using a two-step variable selection process, 11 of these variables were identified for Maxent modeling. Maxent models were developed for seven mosquito species and three time frames: the rainy seasons of 2009 and 2010 combined; November 2009; and November 2010. The importance of environmental variables influencing mosquito distributions was evaluated using percent contributions and permutation importance. Model performance was assessed using the area under the receiver operating characteristic curve (AUROC). AUROC values range from 0 to 1; an AUROC value of 0.5 indicates that the model performs no better than random, an AUROC value of 1 would indicate perfect accuracy, and an AUROC value > 0.8 indicates robust performance of the model [37]. Maxent models are generated using presence-only rather than abundance data; however, because we had abundance data available, we thought it important to test the correlation of Maxent model outputs with both species abundance and CHIKV abundance. Each of these steps is described in greater depth below.

### Data

#### Mosquito data

**Fig. 4:**
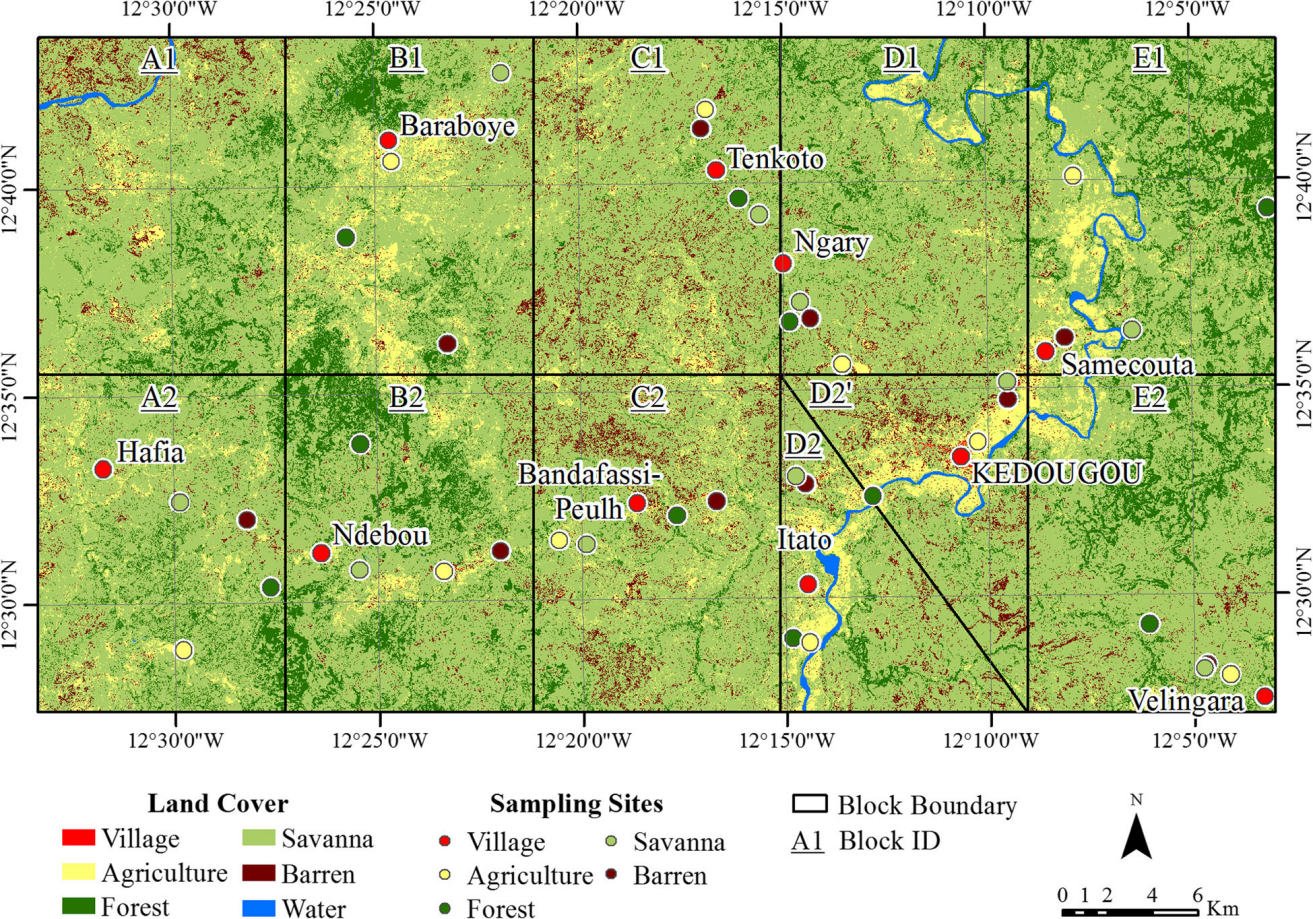
Land cover and location of collection sites in the study area. This is a modified version of the collection site map previously published in [23]. Block A1 was removed from analyses due to inaccessibility; to avoid losing five sampling sites, the most environmentally complex block, Block D2, was subdivided into sub-blocks D2 and D2’ (see [23] for additional information concerning the land cover mapping and mosquito sampling design)

Mosquitoes were collected at 50 sampling sites, including ten in each of five major land cover classes (agricultural land, barren land, forest, savanna and village), as defined by Diallo et al. [23], during the periods of June 2009 to February 2010 and May 2010 to February 2011 (Fig. 4). The 50 sites were chosen using a blocked design and stratified random sampling methods that are described in detail in Diallo et al. [23]. Briefly, we randomly selected three sites for each of the six land cover types in each of ten blocks within one-kilometer buffer zones around roads. The block design eliminated spatial autocorrelation among mosquito observations within a given land cover class. The limitation of sites to fall within a certain distance of roads was necessary to facilitate site accessibility. Three sites per land cover were visited initially to assess whether the land cover on the ground matched the land cover on the map; of the correctly mapped and accessible sites, one was selected randomly for actual sampling. Following the initial site selection process, two of the 50 sampling locations were moved outside of the one-kilometer buffer around roads for logistical reasons.

Mosquitoes were collected using human landing collections, the most effective method for collecting sylvatic *Aedes* species [23, 38–40]. In this study, three people per site collected the mosquitoes that landed on them between 18:00 and 21:00 h. Collections took place monthly for one to four consecutive days. Collectors in the forest were stationed at ground level and on 9 m high platforms; collectors in villages were stationed inside and outside of houses and in the center and periphery of the village. At the end of each collection evening, mosquitoes were frozen and then sorted on a chill-table using morphological identification keys established by Edwards [41], Ferrara et al. [42], Huang [43] and Jupp [44] for the culicines and by Diagne et al. [45] for the anophelines. Identified specimens were sorted into monospecific pools of up to 40 individuals, and tested for CHIKV after cell culture inoculation by RT-PCR.

#### Environmental data

As summarized in Fig. 3, we considered 52 environmental layers from three sources for modeling mosquito species distributions in the study area: a previously derived land cover map [23], Moderate Resolution Imaging Spectroradiometer (MODIS) NDVI 16-Day L3 Global 250m (MOD13Q1; [46]), and WorldClim [47]. These layers were selected for their biological relevance to *Aedes* species distributions [48–51]. All layers were projected to the same spatial reference system (Universal Transverse Mercator, Zone 28P, World Geodetic System 1984) and resampled to 30 m using the nearest neighbor resampling technique to match the finest spatial resolution dataset (i.e. the land cover map) while maintaining the original data values. To maximize model performance using the smallest possible set of uncorrelated predictor variables, we used a multi-step process, which is outlined in the modeling section below. This process reduced the set of 52 initial variables to a set of 18 candidate variables of which three to six were ultimately selected as optimal variables in each of the final species distribution models. Additional file 1: Table S1 lists the complete set of 52 variables as well as the final variables used in the models.

The land cover map (Fig. 4), derived from Landsat 5 Thematic Mapper imagery [23], was used to create five variables relating to the fragmentation and configuration of the landscape at the time of image acquisition, 11 June 2009. These variables thus relate directly to habitat preferences by mosquitoes. The variables included distance to patch edge, patch size, distance from any forest patch, distance from medium to large forest patches (≥ 0.52 km^2^), and distance from large forest patches (≥ 2.14 km^2^). Forest patch size classes used for analysis were determined using Jenk’s natural breaks. All five landscape fragmentation and configuration variables served as candidate variables in the species distribution models (Additional file 1: Table S1).

The MOD13Q1 product was used to derive 25 NDVI variables. The NDVI represents vegetation abundance and thus serves as a proxy measure for cover and nectar available to the mosquitoes, as well as rainfall. MOD13Q1 data are produced on 16-day intervals and were summarized here to capture different NDVI aspects of potential relevance to mosquito habitat selection: maximum, minimum, mean, standard deviation and range of NDVI. To best represent the variation of NDVI across years and seasons, three different time periods were grouped and summarized. The first included all images available for the study period (June 2009 to March 2011), the second all available images for both rainy seasons (July to November 2009/2010), and the third all available images for the two months when the majority of CHIKV isolates were collected (October/November); in this last period we used data from 2009 and 2010 even though CHIKV was only detected in 2009. Of the initial 25 NDVI variables, five were retained as candidate variables in the species distribution models (Additional file 1: Table S1). We recognize that mosquitoes develop over an approximately two-week period and that NDVI during the months preceding the peak of the CHIKV amplification has the potential to be highly predictive of mosquito occurrence. Unfortunately, because of the excessive cloud cover that accompanies the rainy season in Senegal, no NDVI data were available for August and September in either of the rainy seasons included in this study.

WorldClim was used to obtain 22 variables, including 19 bioclimatic and three topographic variables representative of average conditions between 1960 and 1990. Of the 19 bioclimatic variables considered for our model, three (isothermality, precipitation of the driest month and precipitation of the driest quarter) were excluded because they had no or negligible spatial variation in the study area. The remaining 16 bioclimatic variables reflect both the average and extreme temperature and precipitation conditions in the area, both of which have strong influences on mosquito distributions. In addition, the WorldClim altitude layer was used to derive slope and aspect layers and collectively these three serve as indirect measures of microclimate, which also drives mosquito distributions. Of the initial 22 WorldClim variables, five bioclimatic and all three topographic variables were retained as candidate variables in the species distribution models (Additional file 1: Table S1).

### Modeling

#### Rationale for choice of modeling method and of time periods included in the models

Maxent is a machine learning algorithm for modeling species distributions using the principle of maximum entropy in conjunction with species point presence records and environmental raster data. We used Maxent for modeling the distribution of chikungunya vectors because it can create models from as few as five presence points and generally has a similar or better accuracy than other species distribution modeling methods [52–54]. Because multiple basic functions can be used within a single model, it can approximate the complex variable relationships commonly found in ecological data [55]. Additionally, it has been used successfully in multiple studies of the distributions of mosquito vectors of arboviruses and of arboviruses themselves [27, 28, 54, 56–63].

Models used data collected over two general time periods. The first, termed “Two-Year” models, used data collected during all months of the study (June-December) in both years of the study (2009 and 2010) in order to capture overall spatial distributions of each of the seven species. The second, termed “November-Only” models, used data from November, the peak month for the 2009 CHIKV amplification and the only month in the rainy season for which cloud-free imagery could be obtained. Moreover, to detect changes in spatial distributions between the two study years, data from November 2009 and November 2010 were modeled separately.

Although Maxent has been run on samples as small as five, we excluded any model for which there were fewer than ten presence points to improve model accuracy. Moreover, four mosquito species (*Ae. dalzieli*, *Ae. furcifer*, *Ae. luteocephalus* and *Ae. vittatus*) were detected at all 50 sampling sites over the course of the two years, and therefore they were treated as a single unit, termed a “Four-Species Model”, in the Two-Year models. These constraints resulted in a total of 14 models, including four Two-Year models for *Ae. aegypti*, *Ae. africanus*, *Ae. taylori* and the Four Species group; five November-Only models for *Ae. dalzieli*, *Ae. furcifer*, *Ae. luteocephalus*, *Ae. taylori* and *Ae. vittatus* in 2009; and five November-Only models for *Ae. dalzieli*, *Ae. furcifer*, *Ae. luteocephalus*, *Ae. taylori* and *Ae. vittatus* in 2010.

#### Model implementation

We fitted our models using the following settings for background (i.e. locations for which presence was unknown), features (i.e. mathematical transformations of the environmental predictor variables), regularization, sampling bias (i.e. more intense sampling of some environmental conditions than others), model output and model evaluation [64]. The entire study area was chosen as background, because no information was available to justify limiting the geographic or environmental space in which the seven species may occur in the study area. Features were selected automatically as suggested by Phillips & Dudik [55] and further supported by numerous test runs which revealed that, based on their AUROC, models with automatically selected features performed the same or better than models with manually selected features. The default regularization coefficient of 1 was used to select individual features for each predictor following a number of model test runs that showed that both smaller (0.01, 0.1, 0.5) and larger (2, 10) coefficients had higher AUROC values than the default of 1. Sampling bias was taken into account through a bias layer in which a value of 48 was assigned to areas within the original 1 km sampling buffer around roads and a value of 2 to areas outside this buffer; these values represent the number of sampling sites within and outside the buffer, respectively. Each model was run using 15 cross-validation replicates with 75% of samples used for training and 25% used for testing. If there were fewer than 15 data points, the number of replicates equaled the number of points. Predictions from the 15 model runs were averaged to produce final maps of species’ relative occurrence rates, in which a pixel’s value represents the probability that the pixel was included in a collection of presence pixels and in which the values of all pixels in the study area sum to unity [64, 65]. Relative occurrence rates (i.e. Maxent’s raw output) were used as recommended by Merow et al. [64] because they avoid post-processing assumptions. Model performance was evaluated using the AUROC statistic, the most commonly used measure of Maxent model fit [64], despite its potential pitfalls [66, 67].

The relative importance of variables in predicting species distributions was evaluated using jackknife estimates and both percent contributions and permutation importance values. Moreover, to assess how well the models reflected individual species abundance and CHIKV risk, we correlated Maxent model outputs for relative occurrence of a given species (or group of species) at a given site with that species’ abundance (mean females/collector/night) as well as total number of mosquito pools positive for CHIKV (across all species) collected at that site. Correlations were tested using Spearman’s rank correlation, as previous studies have shown that the association between relative occurrence and abundance is often wedge-shaped rather than linear [68].

#### Variable selection

To maximize model performance (maximize AUROC), minimize model overfit (minimize number of variables in model), and minimize multicollinearity (minimize correlation among variables in models), we used a two-step process to select final predictor variables from the initial pool of 52 environmental variables described above. In the first step, we selected 18 candidate variables from the initial 52 using Pearson's correlation coefficients (*r*). From each set of highly cross-correlated (*r* ≥ 0.75) variables, we retained one variable that seemed the most plausible as a biological predictor of mosquito presence and removed all others. In addition, we retained all variables that were not highly cross-correlated (*r* < 0.75). The threshold *r*-value of 0.75 was chosen after Maxent test runs with variables selected based on arbitrary *r* cutoffs of 0.6, 0.75 and 0.9. The *r* threshold of 0.6 was rejected because variables selected using this threshold resulted in models with substantially lower AUROC values than models produced with variables selected using the other *r* thresholds. The *r* threshold of 0.75 was chosen over that of 0.9 because it helped produce models with similar AUROC values while more drastically reducing multicollinearity. In the second step, we used an iterative process for each of the 14 species distribution models to select between three and six optimal variables from the 18 candidate variables for the final models. This process began by running each of the models using the 18 candidate variables, removing the least predictive variable (i.e. variable that most decreased the AUROC), running the models with the new reduced set of predictor variables, removing the next least predictive variable, and so forth, until only variables with predictive power remained.

## Results

### Mosquito species distributions and overall model performance

**Fig. 5:**
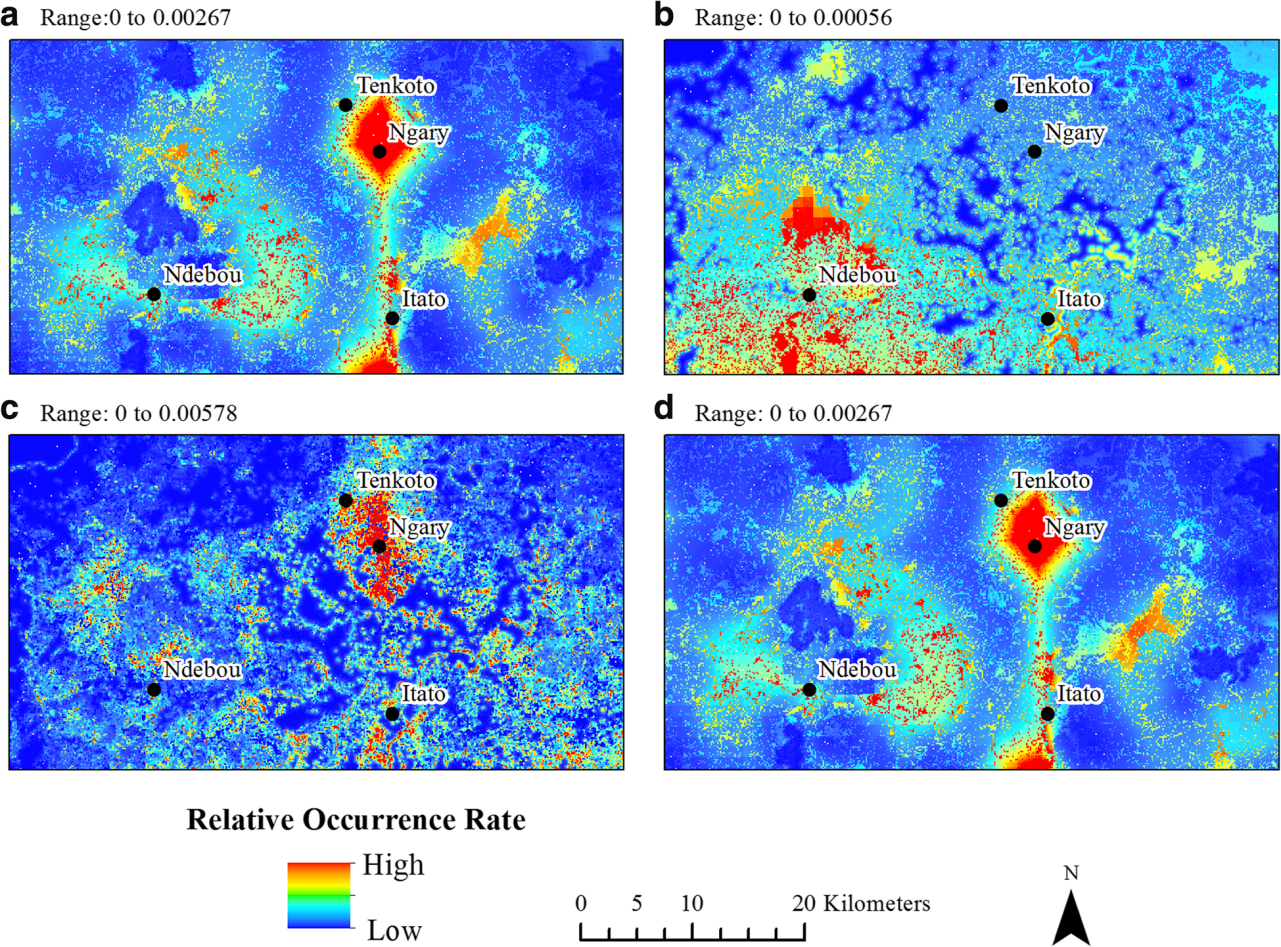
Results of the Two-Year models. **a** *Ae. aegypti.* **b** *Ae. africanus.* **c** *Ae. taylori.* **d** Four-Species model for the four species that were present at all 50 sites (*Ae. dalzieli*, *Ae. furcifer*, *Ae. luteocephalus* and *Ae. vittatus*)

**Fig. 6:**
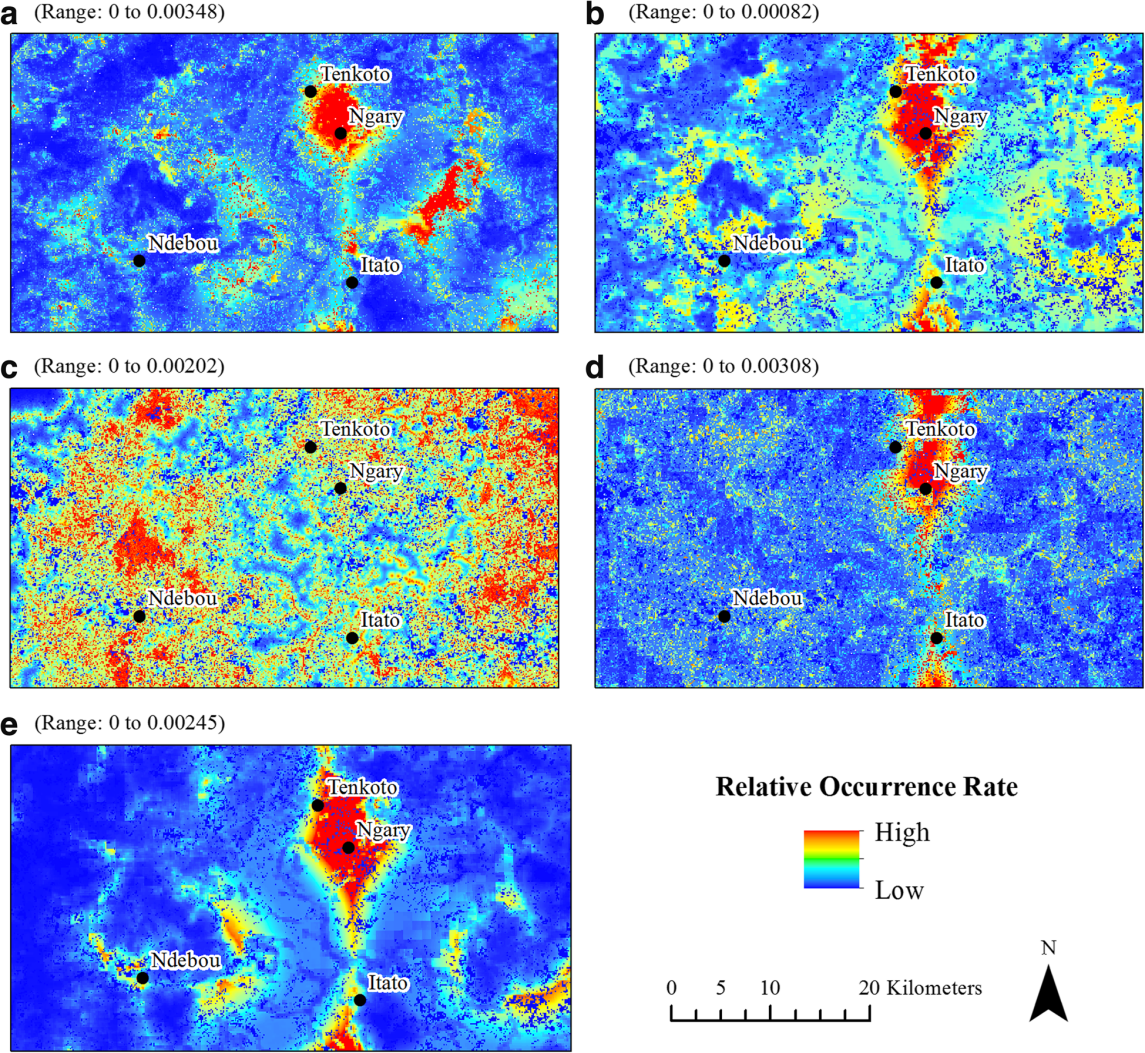
Results of the November-2009-Only models. **a** *Ae. dalzieli.* **b** *Ae. furcifer.* **c** *Ae. luteocephalus.* **d** *Ae. taylori.* **e** *Ae. vittatus. Aedes aegypti* and *Ae. africanus* were detected at fewer than ten sites for this time period and were excluded from analysis

**Fig. 7:**
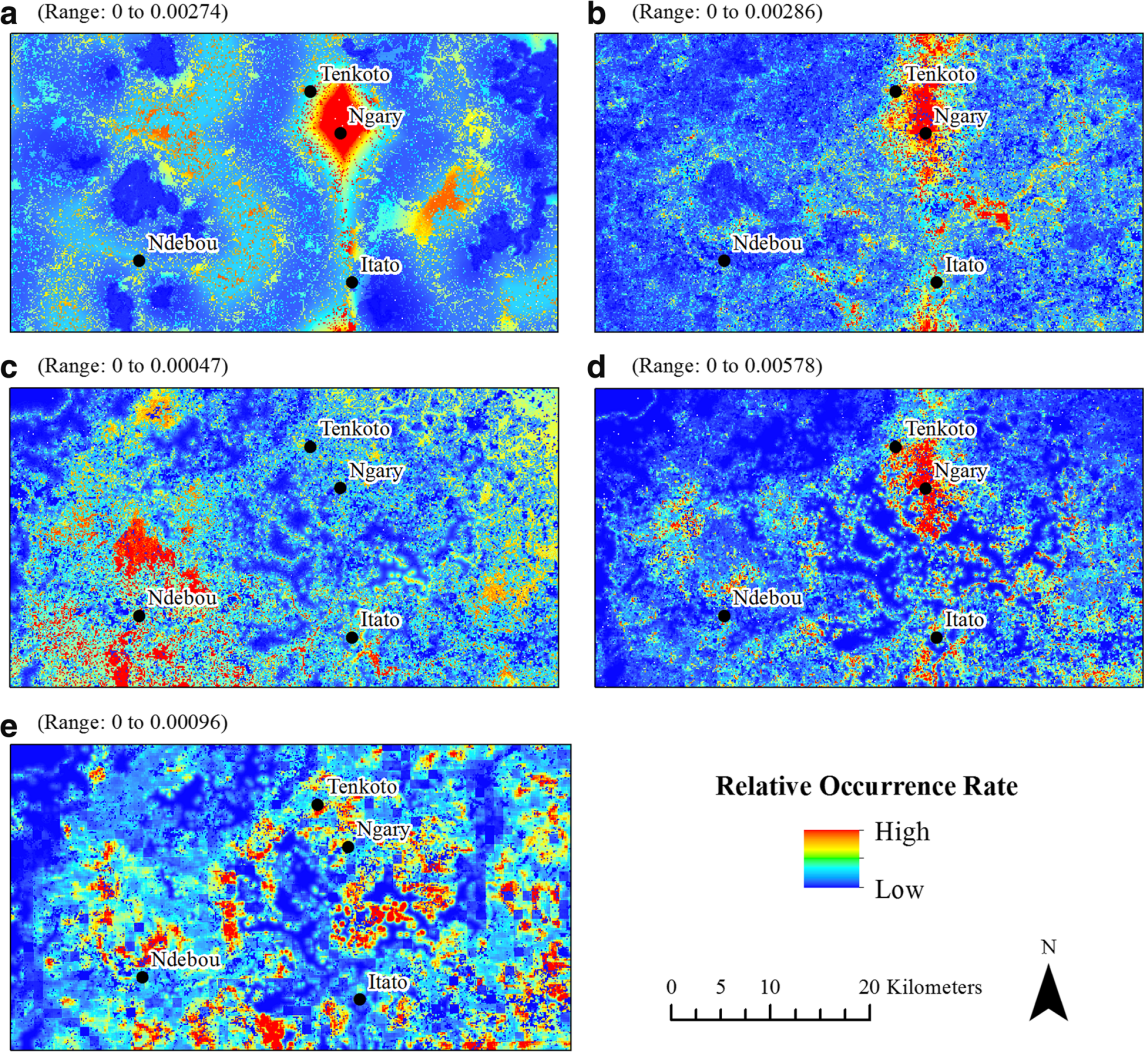
Results of the November-2010-Only models. **a** *Ae. dalzieli*. **b** *Ae. furcifer*. **c** *Ae. luteocephalus*. **d** *Ae. taylori*. **e** *Ae. vittatus. Aedes aegypti* and *Ae. africanus* were detected at fewer than ten sites for this time period and were excluded from analysis

We generated 14 models in total; all models except the November-Only 2009 model for *Ae. furcifer* had AUROC values above 0.7 (Table 1). The relative occurrence rate varied between species and between time periods (Figs. 5, 6 and 7), but three general patterns emerged. First, ten models revealed a pronounced area of high relative mosquito occurrence rates between the villages of Tenkoto and Ngary. These ten models include the Two-Year *Ae. aegypti*, *Ae. taylori* and Four-Species (*Ae. dalzieli*, *Ae. furcifer*, *Ae. luteocephalus* and *Ae. vittatus*) models (Fig. 5a, c and d, respectively); the November-Only *Ae. dalzieli*, *Ae. furcifer*, *Ae. taylori* and *Ae. vittatus* models using data from 2009 (Fig. 6a, b, d and e, respectively); and the November-Only *Ae. dalzieli*, *Ae. furcifer* and *Ae. taylori* models using data from 2010 (Fig. 7a, b and d, respectively). Secondly, five of these ten models with a hotspot around Tenkoto/Ngary also revealed a distinct area of high relative mosquito occurrence rates south of the village of Itato. These five models include the Two-Year *Ae. aegypti* and Four-Species models (Fig. 5a and d, respectively) and the November-Only *Ae. furcifer*, *Ae. taylori* and *Ae. vittatus* models using data from 2009 (Fig. 6b, d and e, respectively). Thirdly, a group of three different models showed a less pronounced though still high relative mosquito occurrence rate north, northeast, and southwest of the village of Ndebou. These three models include the Two-Year *Ae. africanus* model (Fig. 5b) and the November-Only *Ae. luteocephalus* models using data from 2009 and from 2010 (Figs. 6c and 7c, respectively).

**Table 1:** AUROC and correlation values for Maxent models. The average AUROC for each model over fifteen replicates and the standard deviation of those replicates are presented here. Also shown are the Spearman’s rank correlation coefficients (*ρ*) between each model’s predicted relative occurrence rate of a species and (i) that species’ abundance and (ii) the total number of pools positive for CHIKV across all species. *P*-values are listed in parentheses. Boldface text emphasizes statistically significant correlations (*P* < 0.01, α = 0.01 to account for multiple testing)

	Presence points	AUROC	*ρ* Abundance	*ρ* CHIKV
Two Year Models				
*Ae. aegypti*	49	0.720 ± 0.126	0.13 (0.35)	**0.39 (0.005)**
*Ae. africanus*	11	0.769 ± 0.201	**0.45 (0.001)**	0.14 (0.33)
*Ae. taylori*	45	0.682 ± 0.130	-0.22 (0.12)	**0.44 (0.001)**
Four-Species^a^	50	0.723 ± 0.151	Four-Species: 0.02 (0.89) *Ae. dalzieli*: -0.10 (0.51) *Ae. furcifer*: -0.11 (0.47) *Ae.luteocephalus*: -0.04 (0.79) *Ae.vittatus*: -0.10 (0.51)	**0.37 (0.008)**
November 2009 Models				
*Ae. dalzieli*	45	0.744 ± 0.127	0.07 (0.64)	0.32 (0.03)
*Ae. furcifer*	35	0.683 ± 0.102	0.05 (0.71)	0.18 (0.21)
*Ae.luteocephalus*	10	0.727 ± 0.233	**0.45 (0.001)**	0.25 (0.07)
*Ae.taylori*	16	0.773 ± 0.178	**0.51 (0.002)**	**0.56 (< 0.0001)**
*Ae.vittatus*	22	0.820 ± 0.126	**0.67 (0.001)**	0.30 (0.17 )
November 2010 Models				
*Ae. dalzieli*	42	0.746 ± 0.130	0.25 (0.08)	**0.50 (0.0002)**
*Ae. furcifer*	30	0.741 ± 0.136	0.30 (0.04)	**0.43 (0.0002)**
*Ae.luteocephalus*	10	0.782 ± 0.218	**0.47 (0.0007)**	0.24 (0.09)
*Ae.taylori*	17	0.786 ±0.190	**0.63 (< 0.0001)**	**0.45 (0.009)**
*Ae.vittatus*	12	0.811 ±0.120	**0.46 (0.0007)**	0.01 (0.92)

^a^The Four-Species model includes the four species that were present at all 50 sites: *Ae. dalzieli, Ae. furcifer*, *Ae. luteocephalus*, and *Ae. vittatus*. Correlations between the model’s predicted relative occurrence rate and both the four species’ overall combined abundance as well as the individual species’ abundances are shown. To be conservative, we only tested the correlation of the four species together against number of CHIKV pools; however, individual species correlations are shown in the November-Only models

### Environmental factors associated with mosquito presence

The 11 variables used in the final models (Table 2) fall into three groups: (i) indicators of landscape fragmentation (distance from any forest patch, distance from medium to large forest patches, distance from large forest patches, patch size and distance from patch edge); (ii) rainfall/vegetation cover during the rainy season (precipitation of the wettest quarter, mean NDVI for 2009 and 2010 for October and November, range of NDVI for 2009 and 2010 rainy seasons between July and December, range of NDVI for October and November 2009 and 2010); and (iii) topography/microclimate (elevation, slope, aspect). Of the 14 models, all included variables from at least two of these groups and all also used two or more landscape fragmentation variables. Three indicators of landscape fragmentation were the most commonly used explanatory variables: patch size (11 models), distance from large forest patches (ten models), and distance from patch edge (seven models) (see Additional file 1: Table S1 for the range in patch sizes and other variables used in the models and Additional file 2 for the response curves that show how each variable affected the Maxent predictions in each of the models). The mean NDVI for October/November of 2009 and 2010 was the most important precipitation/vegetation variable and included in six models. All other variables were used less frequently and each of the topography/microclimate variables was only relevant in a single model each. Temperature did not explain the distribution of mosquito species in any of the 14 models.

**Table 2:** General magnitude and direction of influence of variables used in the final Maxent models. The numbers indicate the variables’ relative contributions to the final models expressed as a percent contribution (regular numbers) and permutation importance (italicized numbers)

	Two Year Model				November-Only 2009 Model					November-Only 2010 Model				
	*Ae ae*	*Ae af*	*Ae ta*	4S	*Ae da*	*Ae fu*	*Ae lu*	*Ae ta*	*Ae vi*	*Ae da*	*Ae fu*	*Ae lu*	*Ae ta*	*Ae vi*
Distance from any forest patch		-					-					-	-	-
		26.2					27.3					33.9	26.4	23.9
		*30.6*					*35.8*					*42.2*	*39.6*	*21.1*
Distance from medium to large forest patches	cplx			cplx	cplx				cplx	cplx				
	19.5			20.7	22.4				17.2	32.7				
	*30.8*			*31.9*	*34.3*				*33.0*	*48.7*				
Distance from large forest patches	cplx		+	cplx	cplx	+		+	+	cplx	+		+	
	38.8		52.2	38.7	29.2	31.9		39.3	42.9	35.3	36.2		11.6	
	*33.7*		*49.4*	*36.2*	*30.8*	*22.8*		*48.7*	*25.8*	*25.3*	*14.6*		*7.2*	
Distance from patch edge						-	-	-	-		-	-	-	-
						30.3	53.6	17.7	12.2		19.4	24.3	14.9	24.8
						*27.3*	*35.8*	*14.1*	*19.1*		*27.6*	*19.0*	*15.4*	*21.3*
Patch size	-	-		-	-		-	-		-	-	-	-	
	24.8	37.5		23.3	19.2		19.1	32.9		32.0	29.8	33.7	14.6	
	*23.5*	*38.3*		*19.5*	*11.2*		*34.9*	*28.3*		*26.0*	*19.8*	*37.3*	*10.5*	
Mean NDVI for 2009 and 2010 October-November			cplx		+	+			+		+		-	-
			47.8		29.2	37.8			9.4		6.3		12.3	33.1
			*50.6*		*23.7*	*49.9*			*5.8*		*19.8*		*19.2*	*57.5*
Range of NDVI for 2009 and 2010 rainy seasons											-		-	
											8.4		8.4	
											*15.9*		*3.1*	
Precipitation of the wettest quarter	cplx	+		cplx								+	cplx	
	16.9	36.3		17.3								1.4	11.8	
	*12.0*	*31.1*		*12.2*								*8.1*	*5.0*	
Elevation									+					
									18.3					
									*16.3*					
Aspect								+						
								10.1						
								*8.9*						
Slope														+
														18.3
														*0.0*

*Abbreviations*: *Ae ae Ae. aeygpti, Ae af Ae. africanus, Ae da Ae. dalzieli, Ae fu Ae furcifer, Ae lu Ae.luteocephalus, Ae ta Ae. taylori, Ae vi Ae.vittatus*; 4S, Four-Species Model (includes the four species, i.e. *Ae. dalzieli*, *Ae. furcifer*, *Ae. luteocephalus* and *Ae. vittatus*, that were present at all 50 sites)

*Notes*: + indicates a generally positive relationship and - a generally negative relationship between mosquito site suitability and predictor variables; cplx indicates a complex relationship that shifts between positive and negative as the predictor variable value increases

### Correlation of predicted mosquito distributions with mosquito and CHIKV abundance

The Two-Year Four-Species model was neither significantly correlated with the individual abundance of each species in the model (*Ae. dalzieli*, *Ae. furcifer*, *Ae. luteocephalus*, *Ae. vittatus*) nor the overall, combined abundance of the four species (Table 1; see Additional file 3: Tables S2, S3 and S4 for mosquito abundance data for all months, November 2009 and November 2010, respectively). However, the model showed a significant positive correlation with the total number of mosquito pools positive for CHIKV. Of the three species that were modeled individually in Two-Year models (*Ae. aegypti*, *Ae. africanus*, *Ae. taylori*) only the *Ae. africanus* model was significantly correlated with mosquito abundance. However, that model was not correlated with number of CHIKV-positive pools. Both the *Ae. aegypti* and *Ae. taylori* models showed a significant positive correlation with number of CHIKV-positive pools.

Of the ten individual species in November-Only 2009 and November-Only 2010 models, three, i.e. *Ae. luteocephalus*, *Ae. taylori* and *Ae. vittatus*, were significantly correlated with abundance during both years (Table 1). Focusing on 2009, the year of the CHIKV amplification, only the *Ae. taylori* model was correlated with the number of CHIKV-positive mosquito pools. The November-Only 2010 model for *Ae. taylori* was also correlated with number of CHIKV-positive mosquito pools.

## Discussion

Vector-borne disease distributions have been successfully predicted using ecological niche modeling [54, 58, 69–73] and such modeling has led to more effective implementation of vector control methods [74]. In the current study, we used Maxent to model the distribution of seven mosquito vectors of sylvatic CHIKV in a known focus of sylvatic CHIKV transmission in Senegal. While considerable effort has been directed toward modeling distributions of the *Aedes* mosquito vectors of human-endemic CHIKV, dengue virus and Zika virus (e.g. [63, 75, 76]), much less attention has been paid to the distributions of the suite of sylvatic *Aedes* species that maintain the sylvatic cycles of these viruses.

The current study spanned two rainy seasons: 2009, a year in which a CHIKV amplification occurred; and 2010, a year without detectable CHIKV circulation. One group of models utilized data from both rainy seasons combined (Two-Year models) to predict relative occurrence rate of each vector species, while another set of models (November-Only models) utilized data from the one of the months of peak CHIKV transmission (November) from 2009 and 2010 separately. Four of the seven species analyzed (*Ae. dalzieli*, *Ae. furcifer*, *Ae. luteocephalus* and *Ae. vittatus*) were collected from all 50 sites across the two years and their distribution was modeled collectively in the Two-Year models, whereas the remaining three species (*Ae. aegypti*, *Ae. africanus* and *Ae. taylori*) were collected from only a subset of the sites and their distributions were modeled individually. Five of the seven species (*Ae. dalzieli*, *Ae. furcifer*, *Ae. luteocephalus*, *Ae. taylori* and *Ae. vittatus*) were collected in a minimum of ten sites during both November 2009 and 2010 and modeled individually for these months. *Ae. aegypti* in Kédougou comprise the sylvatic subspecies *formosus*, which differs from the urban-dwelling *Ae. aegypti aegypti* in genotype, phenotype, and behavior [77–79].

In combination, these models predicted three distinct spatial hotspots in mosquito distributions. First, *Ae. aegypti*, *Ae. dalzieli*, *Ae. furcifer*, *Ae. luteocephalus*, *Ae. vittatus* and *Ae. taylori* showed high relative occurrence rates in a region between the villages of Tenkoto and Ngary in at least one of the model classes. Secondly, *Ae. aegypti*, *Ae. vittatus*, *Ae. luteocephalus*, *Ae. taylori* and Ae. *dalzieli* had high occurrence rates south of Itato. Finally, *Ae. africanus* and *Ae. luteocephalus* showed high occurrence rates in a region near Ndebou. It is intriguing that *Ae. africanus* showed a distinct distribution from all other species. This suggests that *Ae. africanus* differs in environmental preference from the remaining suite of mosquito vectors. Diallo et al. [23] reported that *Ae. africanus* and *Ae. aegypti formosus* both had a higher abundance in forest than in other land cover classes in Kédougou, but *Ae. africanus* was most abundant in the forest canopy whereas *Ae. aegypti formosus* was most abundant at the forest floor.

The single-species November-Only models also revealed some interesting variation in distributions between the two study years. The distribution of *Ae. taylori* showed a well-defined hotspot near Tenkoto and Ngary in 2009 but a more diffuse hotspot in 2010. The *Ae. vittatus* distribution showed a hotspot near Tenkoto and Ngary in 2009 but no hotspots in 2010. Some of this variation likely reflected changes in rainfall: 2009 was a drier year (94.9 cm of rain) than 2010 (135.9 cm). In contrast, the hotspot in *Ae. luteocephalus*’ distribution was stable between the two years. However, the distribution of *Ae. luteocephalus* was substantially more diffuse than other species distributions in November 2009; this may be because *Ae. luteocephalus* populations decline toward the end of the rainy season, and this decline was particularly abrupt in 2009.

Of the environmental predictors analyzed, landscape fragmentation measures, especially distance to any forest patch, distance to large forest patch, distance from patch edge, and patch size, had particularly large impacts on mosquito occurrence. As we initially anticipated, occurrence of these sylvatic *Aedes* species decreased with increasing distance from a forest patch. The association of mosquitoes with forest patches reflects the sylvatic nature of these species. We previously showed that, during the 2010 amplification of yellow fever virus in Kédougou, villages containing infected mosquitoes pools were significantly closer to large forest patches than villages that did not yield virus-positive pools [25]. Moreover, during the 2011 Zika virus amplification in the region, we detected Zika virus significantly more often in forests than in other land cover classes [80]. In contrast to the current study, models of the anthropophilic vector *Aedes aegypti* have found that this species’ distribution is most closely tied to urban infrastructure [63, 81].

However, and to our surprise, mosquito occurrence generally increased or showed a complex relationship with increasing distance from large forest patches. Complex relationships between these two variables were “U” shaped, with high mosquito occurrence very close to large forest patches, which declined as distance increased but then spiked again at very large distances from large forest patches. The large forest patches in the region were primarily found on mountains, likely because these were inaccessible for forest clearance. Thus, there may be confounding effects between altitude and distance to large forests in this case. Alternatively, collection sites far from large forests may have been in regions of greater fragmentation; as discussed below mosquito occurrence was enhanced at patch edges.

Mosquito occurrence also decreased with increasing distance from patch edge, suggesting that borders between land cover types may represent regions of elevated risk for arbovirus exposure; patch edges have been previously identified as a risk factor for pathogen circulation [82]. Additionally, mosquito occurrence decreased as patch size increased; this is consistent with a positive effect of edge habitat as the edge-interior ratio decreased as patch size increases. These findings indicate that continued fragmentation of the forests in the study region for expansion of activities such as agriculture or mining is likely to increase the risk of sylvatic arbovirus spillover, at least until a time where forest patches of adequate size to support sylvatic *Aedes* species and their hosts are eliminated altogether. Importantly, the vector species modeled here transmit not only sylvatic CHIKV in the region, but also sylvatic dengue virus, yellow fever virus and Zika virus [16]. Thus, our findings provide insight into the environmental risk factors for spillover of all of these viruses.

As we expected, mosquito occurrence generally showed a positive association with variables reflecting precipitation, with the notable exception of *Ae. taylori*, whose occurrence showed a negative or complex relationship with increasing precipitation. The association with precipitation is no surprise given the aquatic larval and pupal stages in the life-cycle of mosquitoes. Schaeffer et al. [83] have previously demonstrated the importance of water dynamics for the distribution of *Ae. africanus* and *Ae. furcifer* in West Africa using mathematical modeling. Moreover, Althouse et al. [16] analyzed the yearly association of weather variables, vector abundance (*Ae. luteocephalus*, *Ae. taylori* and *Ae. furcifer*) and virus abundance in a temporally extensive (1972 and 2008) but spatially limited dataset from Kédougou. Consistent with our findings, they detected an approximately 1% increase in mosquito abundance for each one inch increase in annual rainfall in the region. Interestingly, dengue virus isolations in that study were negatively associated with rainfall, CHIKV isolations were not associated with rainfall, and Zika virus isolations were positively associated with rainfall, revealing the complexity of the transmission dynamics of these viruses and the value of spatially explicit analyses. It is notable in this context that we found that *Ae. taylori* distributions most closely correlated with number of CHIKV positive pools, and that the association between the distribution of this species and precipitation was complex.

Our models did not find temperature to predict occurrence. Temperature is known to be limiting factor for the mosquito life-cycle: *Ae. aegypti* require temperature higher than 10 °C for larval survival [84] and *Ae. vittatus* and *Ae. aegypti* have been found to still be viable following 4.5 months of exposure to 40 °C temperatures [85, 86]. In the current study area, the lowest minimum temperature of the coldest month was 15.9 °C while the highest maximum temperature of the hottest month was 40.2 °C. Thus, the temperatures in the region never exceeded either the lower or the upper threshold for mosquito viability. Temperature also has more subtle effects on rate and success of mosquito development [87, 88], and there was substantial variability in temperature across the study site (Additional file 1: Table S1). However, this variation did not affect probability of occurrence for these species, possibly because of their ability to behaviorally thermoregulate *via* selection of resting sites and oviposition sites.

Having established that mosquito distributions varied across the landscape, we next investigated whether vector occurrence was correlated with species abundance. Previous studies, e.g. [68, 89, 90] have had mixed success in using Maxent predictions of ecological suitability of a species to predict that species’ abundance. In this study we also found considerable variation in the correlation between model predictions of relative occurrence and empirical measures of species abundance obtained *via* human landing collections; a significant correlation was obtained in only half of the fourteen comparisons.

Our overarching research goal is to predict and control CHIKV spillover [91, 92]. To achieve this goal it is critical to be able to predict the distribution of CHIKV across the landscape. To this end, we found that the distribution of one species, *Ae. taylori*, was strongly correlated with the presence of CHIKV in the Two-Year model, the November-2009 model, and the November-2010 model. Although the distribution of this species was most closely associated with CHIKV detection, this does not necessarily imply that it is the major vector of this virus. Nonetheless, we have shown that in 2009 *Ae. taylori* had the highest infection rate with CHIKV of the seven mosquito species considered in this study [23]. Diallo et al. [23] implicated *Ae. taylori* as a vector of the sylvatic cycle of CHIKV within wildlife but suggested that *Ae. furcifer* was the most likely vector of CHIKV spillover into humans due to this species’ broad distribution and high parity in villages.

It must be emphasized that vector distribution models reflect the potential rather than the comprehensive and complete distribution of vector [93, 94]. Nonetheless, vector models are usually the most effective way to model vector-borne pathogens because vectors have more limited dispersal ranges than their hosts. Attempts have been made to model vector-borne pathogen distributions using human cases of disease (e.g. [95, 96]), but the true distribution of pathogens in these models may be obscured by the high mobility of humans, misdiagnosis of the disease, and asymptomatic infection. Modeling based on the presence of non-human hosts is even more difficult, as it requires extensive trapping of wild vertebrate animals. Our study demonstrates that hotspots of presence of a particular vector species is indeed correlated with detection of virus in those areas. By integrating vector, virus and environmental data in spatial models, studies such as this facilitate disease risk analysis and the development and improvement of vector and virus monitoring and control efforts [97–99].

## Conclusions

We generated ecological niche models for the *Aedes* mosquito vectors of four sylvatic mosquito-borne viruses (CHIKV, dengue virus, yellow fever virus, and Zika virus [92]) in Kédougou, Senegal. These models revealed three key environmental factors, i.e. proximity to large patches of forest, land cover patch size and precipitation, that are strongly associated with the presence of these vectors. Moreover, the distributions of one vector species, *Ae. taylori*, was highly correlated with detection of CHIKV. It should therefore be possible to utilize data on the environmental factors and vector distributions listed above to predict the location of future CHIKV amplifications in the region.

## Additional files

Additional file 1: **Table S1.** Environmental layers considered for use in the mosquito species distribution models. A list of variables used in the models, their description, source, native spatial resolution and data range. (DOCX 20 kb)

Additional file 2: Maxent response curves for the mosquito habitat suitability models. A compilation of figures that show how the environmental variables affected the Maxent predictions. The red curve in each figure shows the mean response of the fifteen replicate Maxent runs; the blue shaded areas indicate the mean ± one standard deviation. The x-axis represents the variable value; the y-axis represents the relative occurrence rate. (DOCX 810 kb)

Additional file 3: **Table S2.** Abundance of mosquito species for all months. **Table S3.** Abundance of mosquito species for November 2009. **Table S4.** Abundance of mosquito species for November 2010. Abundance is the number of mosquitoes per collector per night of collection and reported per collection site. Eastings and northings are in UTM coordinates, Zone 28P, WGS 1984. (XLSX 27 kb)

## Abbreviations

AUROC: Area under the receiver operating characteristic curve
CHIKV: Chikungunya virus
MODIS: Moderate resolution imaging spectroradiometer
NDVI: Normalized difference vegetation index
RT-PCR: Reverse transcription polymerase chain reaction
