# Supplementary material for: Ecological niche modeling of Aedes mosquito vectors of chikungunya virus in southeastern Senegal
Source: Parasit Vectors. 2018 Apr 19;11:255. doi: 10.1186/s13071-018-2832-6 (PMC5907742; doi:10.1186/s13071-018-2832-6)
Supplement: Supplementary file 1 — Table S1. Environmental layers considered for use in the mosquito species distribution models. A list of variables used in the models, their description, source, native spatial resolution and data range. (DOCX 20 kb) [file 13071_2018_2832_MOESM1_ESM.docx]

**Additional file 1: Table S1.**  **Environmental layers considered for use in the mosquito species distribution models.**

| \| **Name** \| **Explanation** \| **Data Source^1^** \| **Native Spatial Resolution** \| **Data Range in Study Area** \| \| --- \| --- \| --- \| --- \| --- \| \| *DistForest*^2,3^ \| Distance from any forest patch \| Land Cover \| 30 m \| 0.0 – 1,336.6 m \| \| *DistForestMed*^2,3^ \| Distance from medium to large (≥ 0.52 km^2^) forest patches \| Land Cover \| 30 m \| 0.0 – 12,756.9 m \| \| *DistForestLarge*^2,3^ \| Distance from large (≥ 2.14 km^2^) forest patches \| Land Cover \| 30 m \| 0.0- 17,406.3 m \| \| *DistEdge*^2,3^ \| Distance from patch edge \| Land Cover \| 30 m \| 0.0 - 268.3 m \| \| *PatchSize*^2,3^ \| Patch size \| Land Cover \| 30 m \| 576.3 - 1.2 x10^9^ m^2^ \| \| *NDVIMin*^2^ \| Minimum NDVI, June 2009 to March 2011 \| MOD13Q1 \| 250 m \| 0.137 - 0.336 \| \| *NDVIMax* \| Maximum NDVI, June 2009 to March 2011 \| MOD13Q1 \| 250 m \| 0.340-0.864 \| \| *NDVIMean* \| Mean NDVI, June 2009 to March 2011 \| MOD13Q1 \| 250 m \| 0.210-0.565 \| \| *NDVIRan* \| Range of NDVI, June 2009 to March 2011 \| MOD13Q1 \| 250 m \| 0.177-0.662 \| \| *NDVIStd* \| Standard deviation of NDVI, June 2009 to March 2011 \| MOD13Q1 \| 250 m \| 0.055-0.208 \| \| *NDVIMinWet* \| Minimum NDVI, July-November 2009/2010 \| MOD13Q1 \| 250 m \| 0.179-0.635 \| \| *NDVIMaxWet* \| Maximum NDVI, July-November 2009/2010 \| MOD13Q1 \| 250 m \| 0.340-0.864 \| \| *NDVIMeanWet* \| Mean NDVI, July-November 2009/2010 \| MOD13Q1 \| 250 m \| 0.264-0.717 \| \| *NDVIRanWet*^2^ \| Range of NDVI, July-November 2009/2010 \| MOD13Q1 \| 250 m \| 0.080-0.549 \| \| *NDVIStdWet* \| Standard deviation of NDVI, July-November 2009/2010 \| MOD13Q1 \| 250 m \| 0.023-0.176 \| \| *NDVIMinON09* \| Minimum NDVI, October/November 2009 \| MOD13Q1 \| 250 m \| 0.234-0.758 \| \| *NDVIMaxON09* \| Maximum NDVI, October/November of 2009 \| MOD13Q1 \| 250 m \| 0.340-0.864 \| \| *NDVIMeanON09* \| Mean NDVI, October/November 2009 \| MOD13Q1 \| 250 m \| 0.298-0.800 \| \| *NDVIRanON09* \| Range of NDVI, October/November of 2009 \| MOD13Q1 \| 250 m \| 0.001-.0374 \| \| *NDVIStdON09* \| Standard deviation of NDVI, October/November 2009 \| MOD13Q1 \| 250 m \| 0.000-0.159 \| \| *NDVIMinON10* \| Minimum NDVI, October/November 2010 \| MOD13Q1 \| 250 m \| 0.237-0.738 \| \| *NDVIMaxON10* \| Maximum NDVI, October/November 2010 \| MOD13Q1 \| 250 m \| 0.275-0.784 \| \| *NDVIMeanON10* \| Mean NDVI, October/November 2010 \| MOD13Q1 \| 250 m \| 0.259-0.758 \| \| *NDVIRanON10*^2^ \| Range of NDVI, October/November 2010 \| MOD13Q1 \| 250 m \| 0.000-0.293 \| \| *NDVIStdON10* \| Standard deviation of NDVI, October/November 2010 \| MOD13Q1 \| 250 m \| 0.000-0.147 \| \| *NDVIMinON0910* \| Minimum NDVI, October/November 2009/2010 \| MOD13Q1 \| 250 m \| 0.234-0.720 \| \| *NDVIMaxON0910* \| Maximum NDVI, October/ November 2009/2010 \| MOD13Q1 \| 250 m \| 0.340-0.864 \| \| *NDVIMeanON0910*^2,3^ \| Mean NDVI, October/November 2009/2010 \| MOD13Q1 \| 250 m \| 0.289-0.773 \| \| *NDVIRanON0910*^2,3^ \| Range of NDVI, October/November 2009/2010 \| MOD13Q1 \| 250 m \| 0.022-0.388 \| \| *NDVIStdON0910* \| Standard deviation of NDVI, October/November 2009/2010 \| MOD13Q1 \| 250 m \| 0.008-0.136 \| \| *ATemp*^2^ \| Annual mean temperature \| WorldClim \| 30” \| 26.2-28.6 C° \| \| *APrecip* \| Annual precipitation \| WorldClim \| 30” \| 1,131-1,272 mm \| \| *MaxTWarmM* \| Maximum temperature of the warmest month \| WorldClim \| 30” \| 37.1-40.2 C° \| \| *Isothermality* \| Mean diurnal temperature range divided by annual temperature range * 100 \| WorldClim \| 30” \| 56-58 \| \| *MeanDiurnal* \| Mean diurnal range (mean of monthly (max temp - min temp)) \| WorldClim \| 30” \| 12.3-13.3 C° \| \| *MeanTColdQ* \| Mean temperature of coldest quarter \| WorldClim \| 30” \| 24.1-26.4 C° \| \| *MeanTDryQ* \| Mean temperature of driest quarter \| WorldClim \| 30” \| 26.4-29.0 C° \| \| *MeanTWarmQ* \| Mean temperature of warmest quarter \| WorldClim \| 30” \| 29.6-32.3 C° \| \| *MeanTWetQ* \| Mean temperature of wettest quarter \| WorldClim \| 30” \| 24.7-27.1 C° \| \| *MinTColdM* \| Minimum temperature of coldest month \| WorldClim \| 30” \| 15.9-17.3 C° \| \| *PrecipColdQ* \| Precipitation of coldest quarter \| WorldClim \| 30” \| 6-12 mm \| \| *PrecipSeason*^2^ \| Precipitation seasonality (coefficient of variation) \| WorldClim \| 30” \| 117 -121 \| \| *PrecipDryM* \| Precipitation of driest month \| WorldClim \| 30” \| 0 mm \| \| *PrecipDryQ* \| Precipitation of driest quarter \| WorldClim \| 30” \| 0 mm \| \| *PrecipWarmQ*^2^ \| Precipitation of wettest quarter \| WorldClim \| 30” \| 49-62 mm \| \| *PrecipWetM* \| Precipitation of wettest month \| WorldClim \| 30” \| 309-339 mm \| \| *PrecipWetQ*^2,3^ \| Precipitation of wettest quarter \| WorldClim \| 30” \| 824-895 mm \| \| *TempRange* \| Annual temperature range (*MaxTWarmM* -*MinTColdM*) \| WorldClim \| 30” \| 21.2-23.0 C° \| \| *TempSeason*^2^ \| Temperature seasonality (standard deviation *100) \| WorldClim \| 30” \| 245.8 – 221.2 \| \| *Elevation*^2,3^ \| Elevation \| WorldClim \| 30” \| 80-463 m \| \| *Aspect*^2,3^ \| Direction slope is facing, expressed as northness \| WorldClim \| 30” \| -1-1 \| \| *Slope*^2,3^ \| Slope \| WorldClim \| 30” \| 1.35-87.10° \| |
| --- | --- | --- | --- | --- | --- | --- | --- | --- | --- | --- | --- | --- | --- | --- | --- | --- | --- | --- | --- | --- | --- | --- | --- | --- | --- | --- | --- | --- | --- | --- | --- | --- | --- | --- | --- | --- | --- | --- | --- | --- | --- | --- | --- | --- | --- | --- | --- | --- | --- | --- | --- | --- | --- | --- | --- | --- | --- | --- | --- | --- | --- | --- | --- | --- | --- | --- | --- | --- | --- | --- | --- | --- | --- | --- | --- | --- | --- | --- | --- | --- | --- | --- | --- | --- | --- | --- | --- | --- | --- | --- | --- | --- | --- | --- | --- | --- | --- | --- | --- | --- | --- | --- | --- | --- | --- | --- | --- | --- | --- | --- | --- | --- | --- | --- | --- | --- | --- | --- | --- | --- | --- | --- | --- | --- | --- | --- | --- | --- | --- | --- | --- | --- | --- | --- | --- | --- | --- | --- | --- | --- | --- | --- | --- | --- | --- | --- | --- | --- | --- | --- | --- | --- | --- | --- | --- | --- | --- | --- | --- | --- | --- | --- | --- | --- | --- | --- | --- | --- | --- | --- | --- | --- | --- | --- | --- | --- | --- | --- | --- | --- | --- | --- | --- | --- | --- | --- | --- | --- | --- | --- | --- | --- | --- | --- | --- | --- | --- | --- | --- | --- | --- | --- | --- | --- | --- | --- | --- | --- | --- | --- | --- | --- | --- | --- | --- | --- | --- | --- | --- | --- | --- | --- | --- | --- | --- | --- | --- | --- | --- | --- | --- | --- | --- | --- | --- | --- | --- | --- | --- | --- | --- | --- | --- | --- | --- | --- | --- | --- | --- | --- | --- | --- | --- | --- | --- | --- | --- | --- | --- | --- | --- | --- | --- | --- | --- |

^1^ Data sources: land cover map (representative of conditions on 11 June 2009; Diallo et al. 2012), MOD13Q1 (representative of conditions between June 2009 and March 2011; NASA Land Processes Distributed Active Archive Center 2012), WorldClim (representative of average conditions between 1960-1990; Hijmans et al. 2005).

^2^ Candidate variables

^3^ Optimal variables

References Cited

Diallo, D., A. A. Sall, M. Buenemann, R. Chen, O. Faye, C. T. Diagne, O. Faye, Y. Ba, I. Dia, D. Watts, S. C. Weaver, K. A. Hanley, and M. Diallo. 2012. Landscape ecology of sylvatic chikungunya virus and mosquito vectors in southeastern Senegal. *PLoS Neglected Tropical Diseases* 6 (6): e1649.

Hijmans, R. J., S. E. Cameron, J. L. Parra, P. G. Jones, and A. Jarvis. 2005. Very high resolution interpolated climate surfaces for global land areas. *International Journal of Climatology* 25 (15): 1965-1978.

NASA Land Processes Distributed Active Archive Center. 2012. *MODIS NDVI 16-Day L3 Global 250m, MOD13Q1*. USGS/Earth Resources Observation and Science (EROS) Center 2010 [cited 1 August 2012]. Available from https://lpdaac.usgs.gov/dataset_discovery/modis/modis_products_table/mod13q1.
