# Supplementary material for: Ecological niche modeling of Aedes mosquito vectors of chikungunya virus in southeastern Senegal
Source: Parasit Vectors. 2018 Apr 19;11:255. doi: 10.1186/s13071-018-2832-6 (PMC5907742; doi:10.1186/s13071-018-2832-6)
Supplement: Supplementary file 2 — Maxent response curves for the mosquito habitat suitability models. A compilation of figures that show how the environmental variables affected the Maxent predictions. The red curve in each figure shows the mean response of the fifteen replicate Maxent runs; the blue shaded areas indicate the mean ± one standard deviation. The x-axis represents the variable value; the y-axis represents the relative occurrence rate. (DOCX 810 kb) [file 13071_2018_2832_MOESM2_ESM.docx]

**Additional file 2.**  **Maxent response curves for the mosquito habitat suitability models.**

The figures below show how the environmental variables affected the Maxent predictions. The red curve in each figure shows the mean response of the fifteen replicate Maxent runs; the blue shade areas indicate the mean +/- one standard deviation. The x-axis represents the variable value; the y-axis represents the relative occurrence rate.

Two-Year Models

*Ae. aeygpti* (page 2)

*Ae. africanus* (page 3)

*Ae. taylori* (page 4)

Four-Species (page 5)

November-Only 2009 Model

*Ae. dalzieli* (page 6)

*Ae. furcifer* (page 7)

*Ae.luteocephalus* (page 8)

*Ae.taylori* (page 9)

*Ae.vittatus* (page 10)

November-Only 2009 Model

*Ae. dalzieli* (page 11)

*Ae. furcifer* (page 12)

*Ae.luteocephalus* (page 13)

*Ae.taylori* (page 14)

*Ae.vittatus* (page 15)

***Aedes aegypti*, Two-Year Model**

| Distance from Large Forest Patches  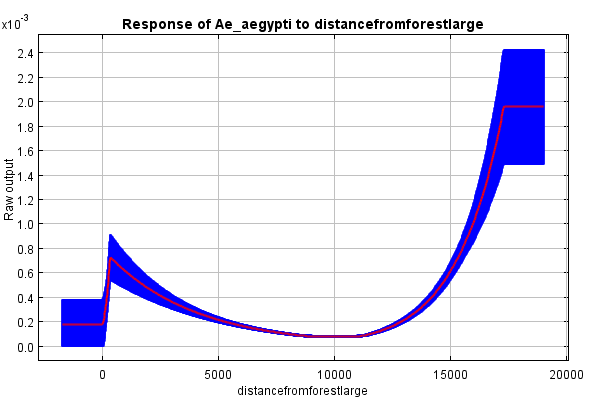 | Distance from Medium to Large Forest Patches  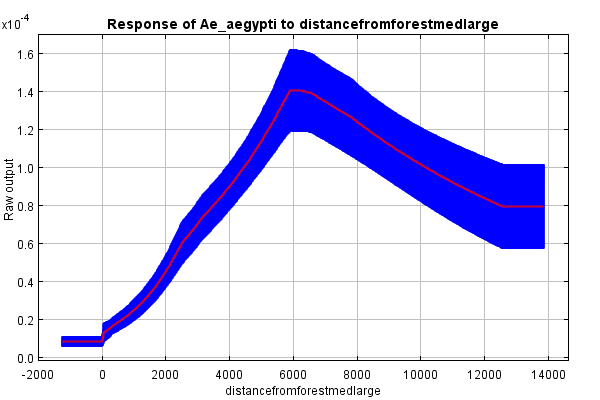 |
| --- | --- |
| Patch Size  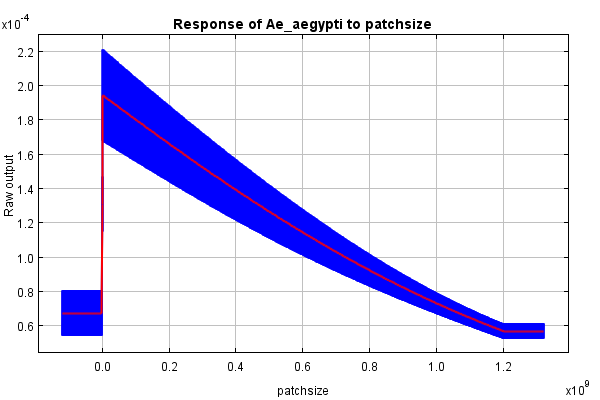 | Precipitation of the Wettest Quarter  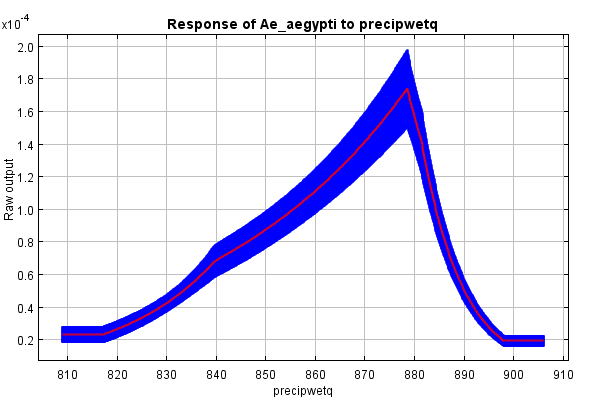 |

***Aedes africanus,* Two-Year Model**

| Distance from Any Forest Patch 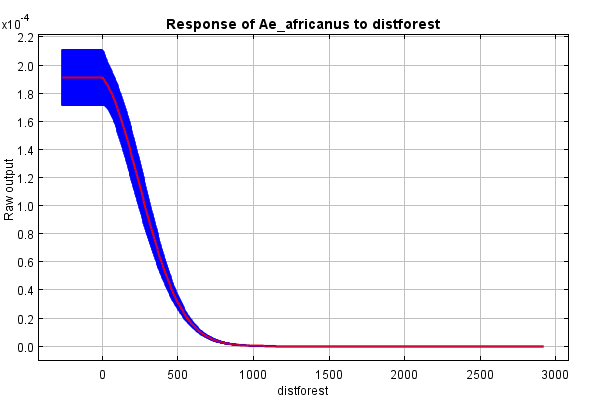 | Patch Size  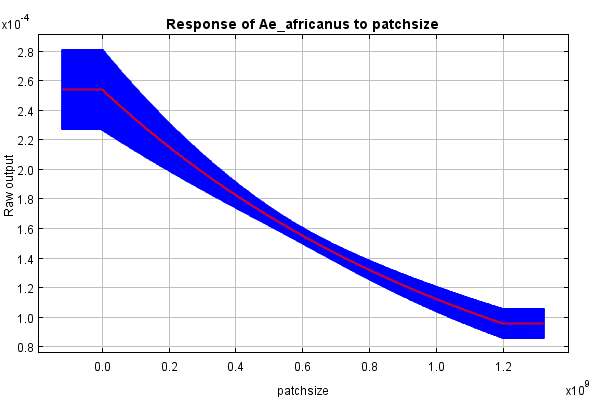 |
| --- | --- |
| Precipitation of the Wettest Quarter  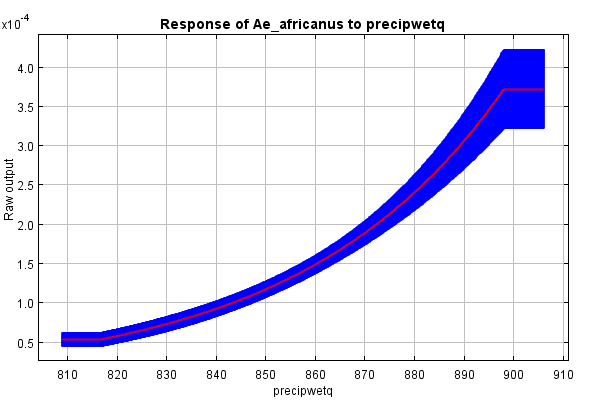 |  |

***Aedes taylori*, Two-Year Model**

| Mean NDVI for 2009 and 2010 October-November 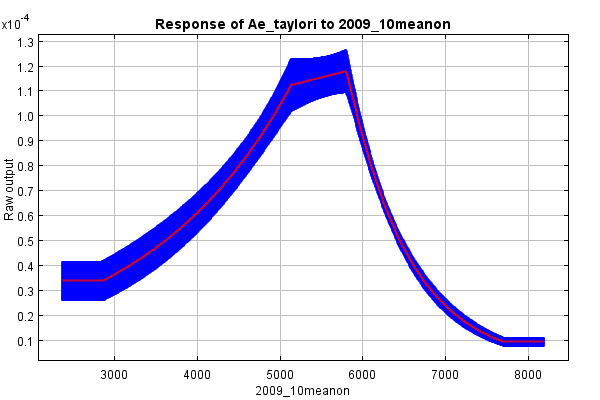 | Distance from Large Forest Patches 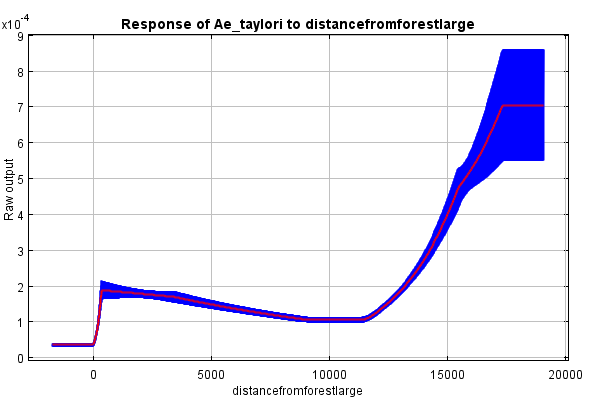 |
| --- | --- |

**All Species, Two-Year Model**

| Distance from Large Forest Patches  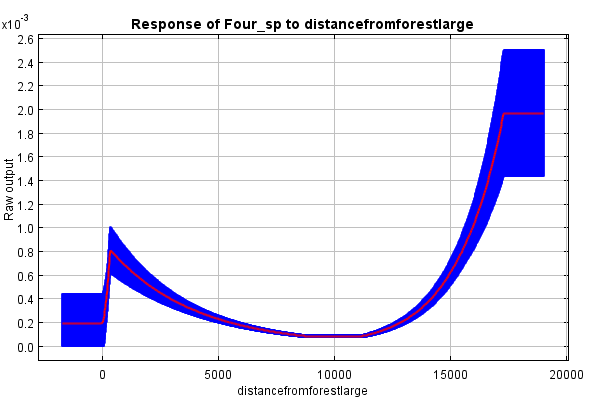 | Distance from Medium to Large Forest Patches  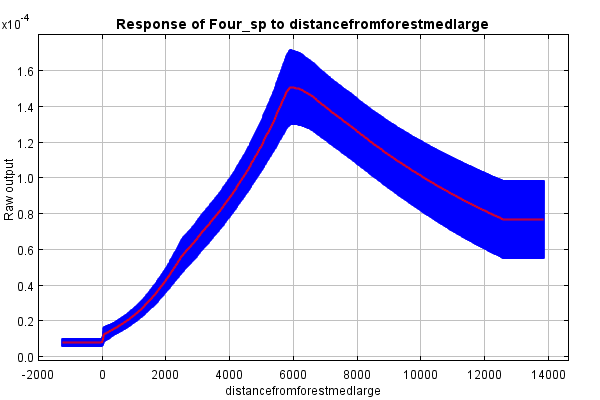 |
| --- | --- |
| Patch Size  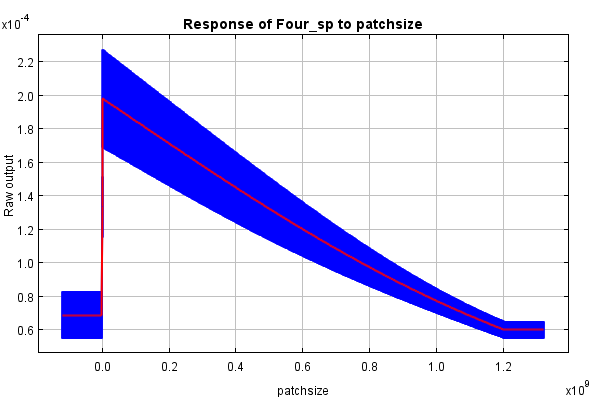 | Precipitation of the Wettest Quarter  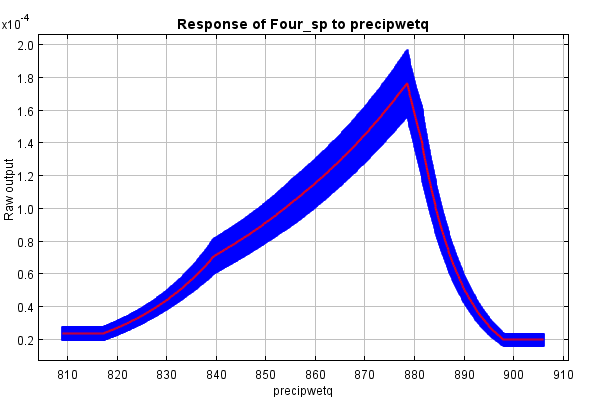 |

***Ae. dalzieli,* November-Only 2009 Model**

| Mean NDVI for 2009 and 2010 October-November *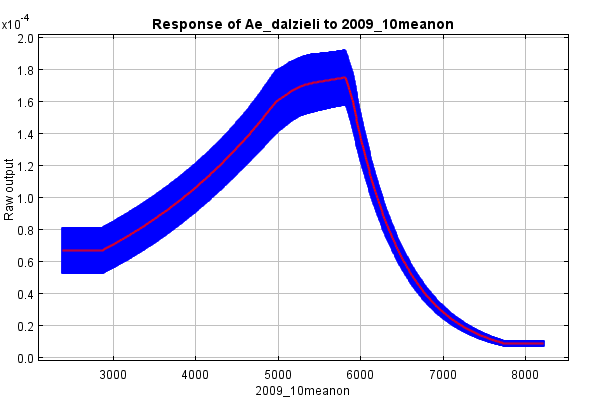* | Distance from Medium to Large Forest Patches  *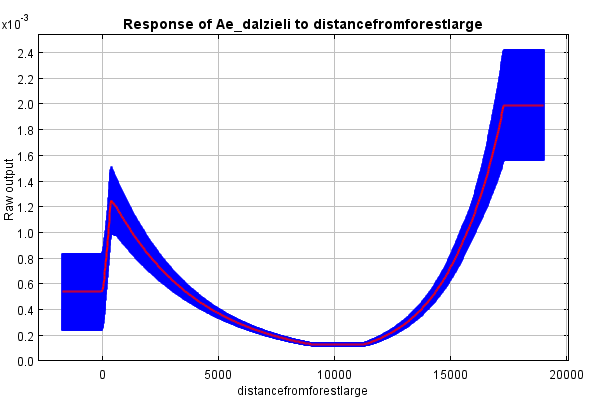* |
| --- | --- |
| Distance from Medium to Large Forest Patches *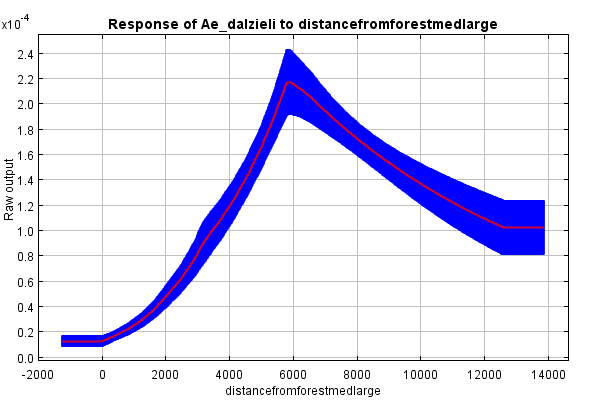* | Patch Size  *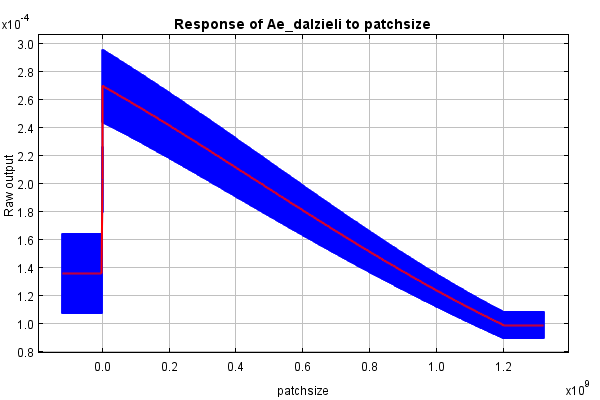* |

***Ae. furcifer,* November-Only 2009 Model**

| Mean NDVI for 2009 and 2010 October-November  *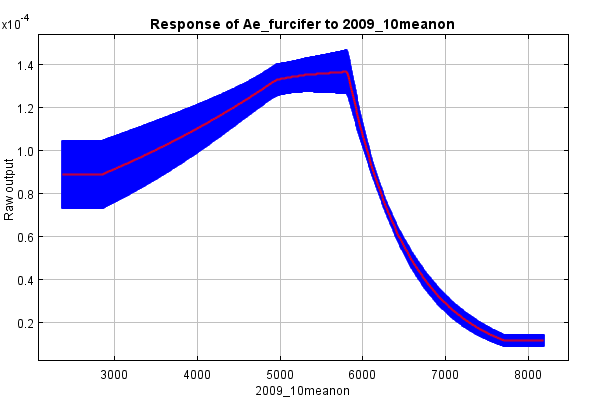* | Distance from Large Forest Patches  *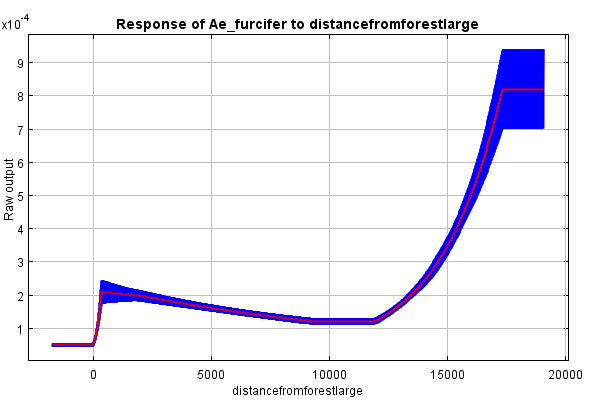* |
| --- | --- |
| Distance From Patch Edge  *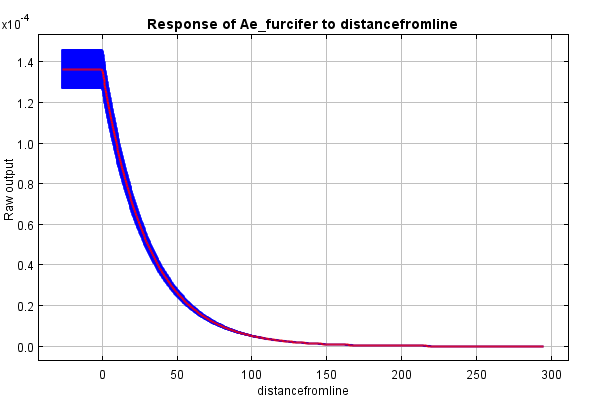* |  |

***Ae.luteocephalus,* November-Only 2009 Model**

| Distance From Patch Edge  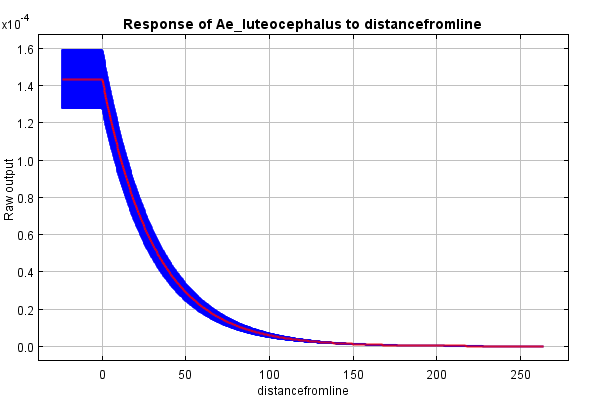 | Distance from Any Forest Patch  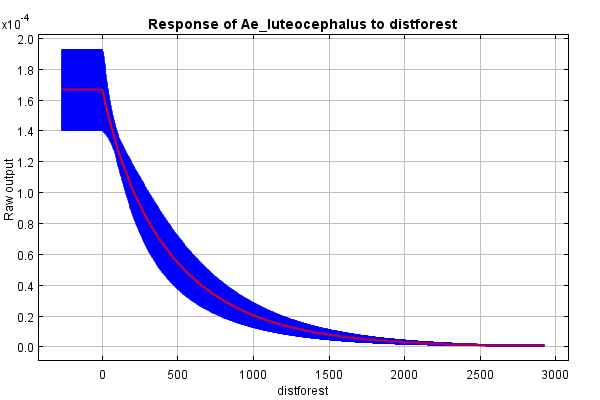 |
| --- | --- |
| Patch Size  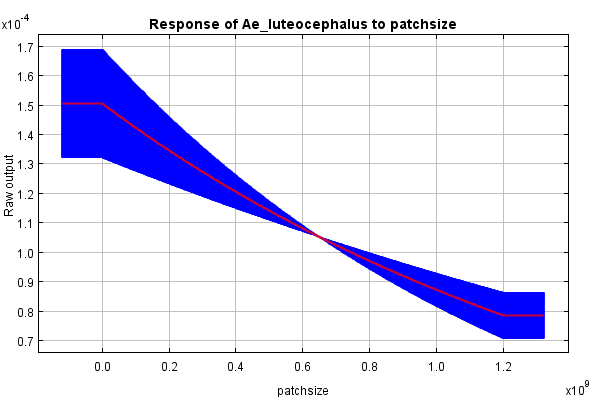 |  |

***Ae.taylori,* November-Only 2009 Model**

| Aspect  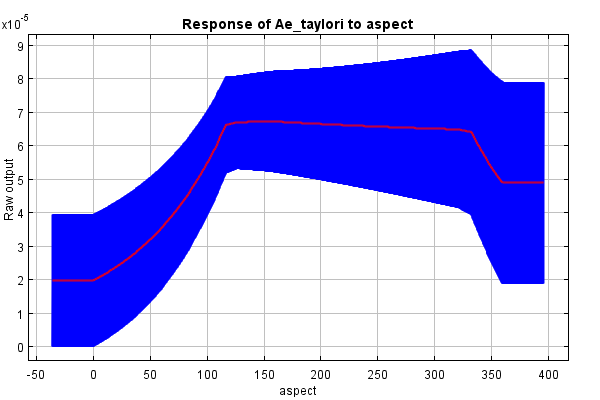 | Distance from Medium to Large Forest Patches  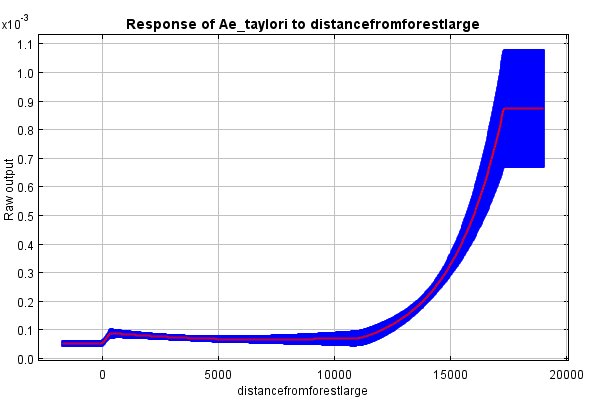 |
| --- | --- |
| Distance From Patch Edge  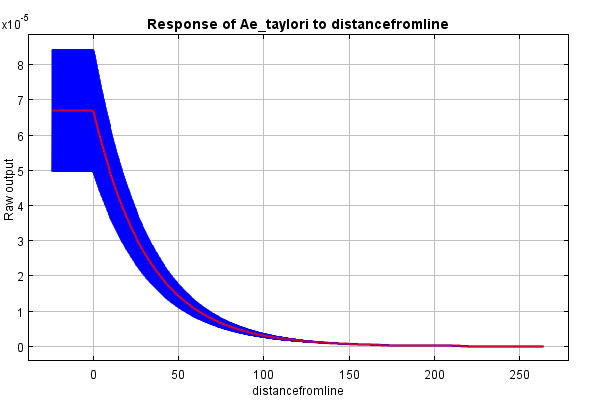 | Patch Size  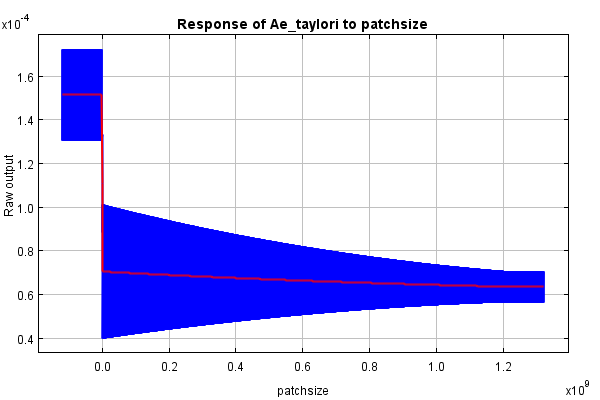 |

***Ae.vittatus,* November-Only 2009 Model**

| Mean NDVI for 2009 and 2010 October-November  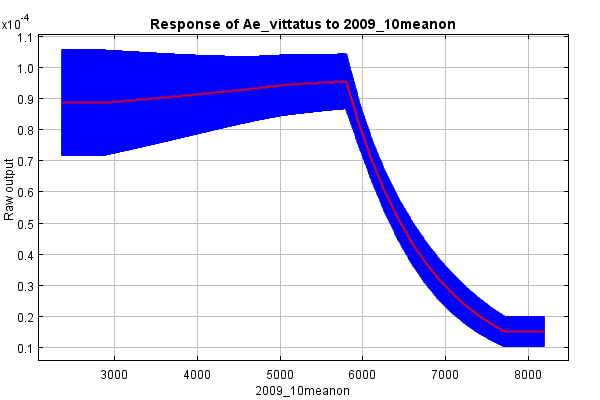 | Distance from Medium to Large Forest Patches  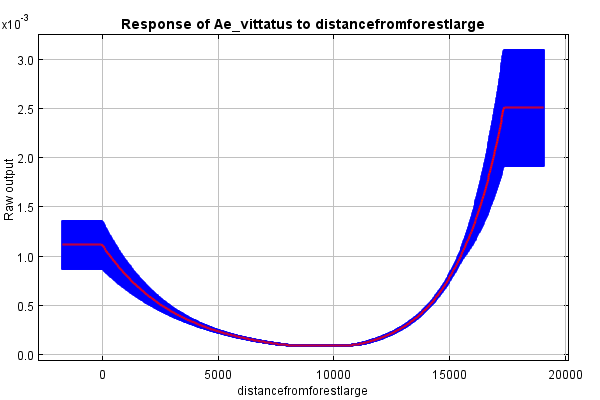 |
| --- | --- |
| Distance from Large Forest Patches  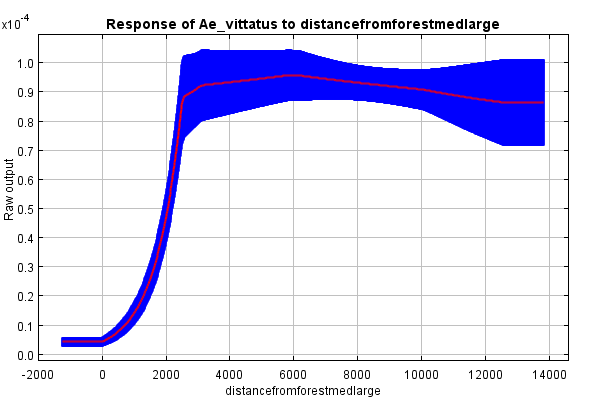 | Distance From Patch Edge  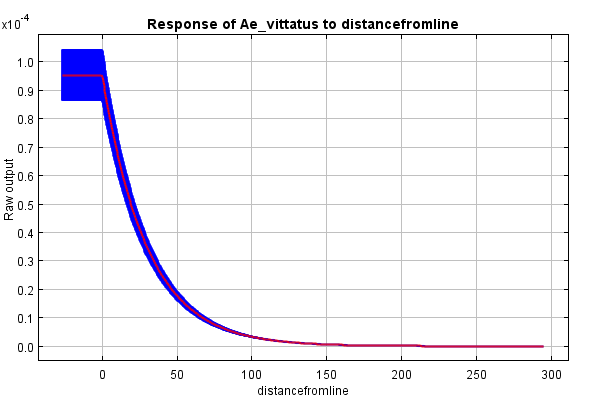 |
| Elevation  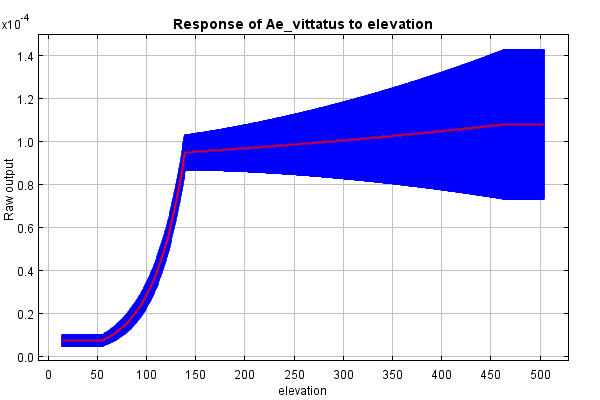 |  |

***Ae. dalzieli,* November-Only 2010 Model**

| Distance from Large Forest Patches  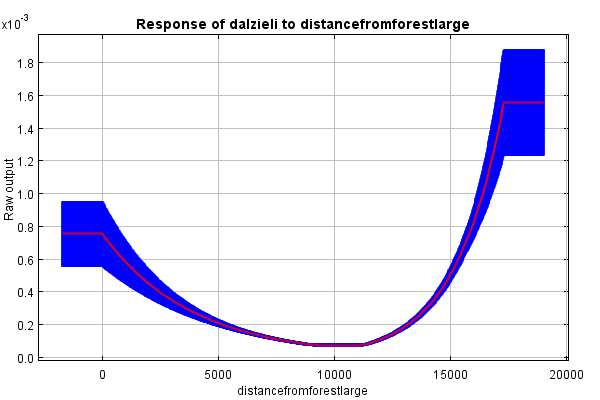 | Distance from Medium to Large Forest Patches  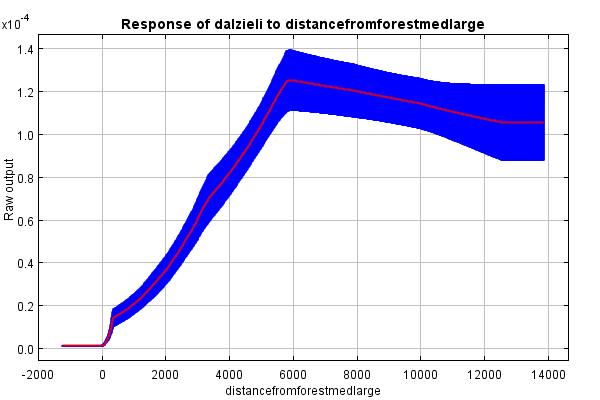 |
| --- | --- |
| Patch Size  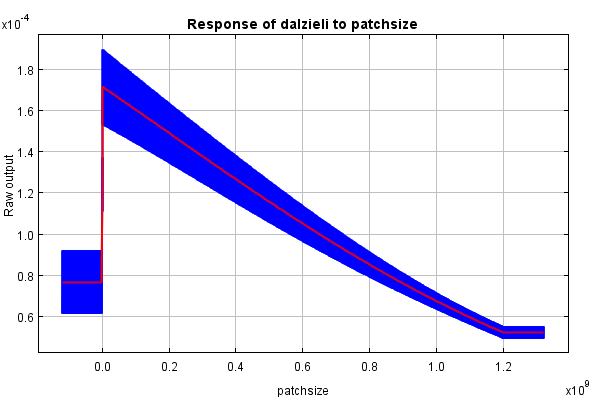 |  |

***Ae. furcifer,* November-Only 2010 Model**

| Mean NDVI for 2009 and 2010 October-November  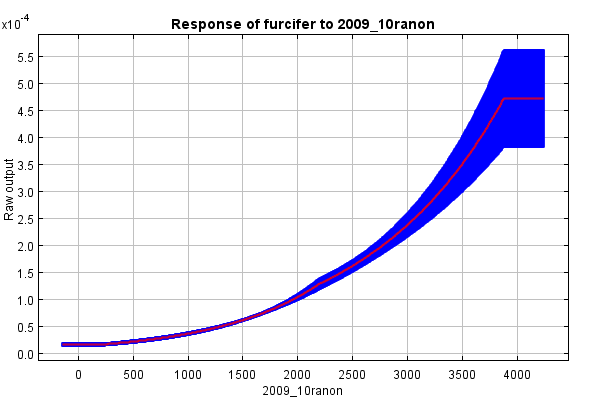 | Distance from Large Forest Patches 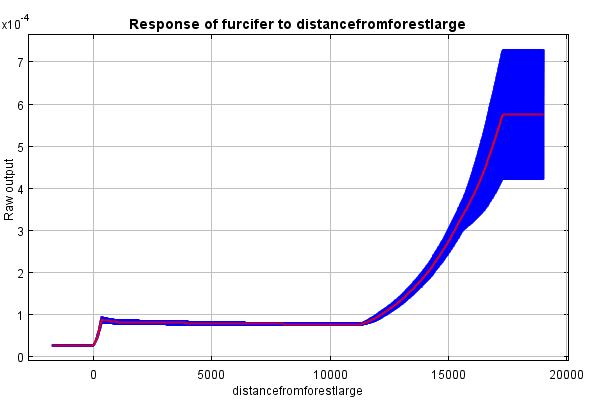 |
| --- | --- |
| Distance From Patch Edge  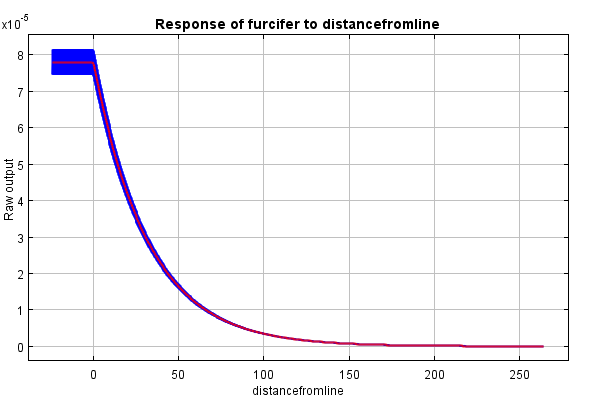 | Range of NDVI for 2009 and 2010 Rainy Seasons  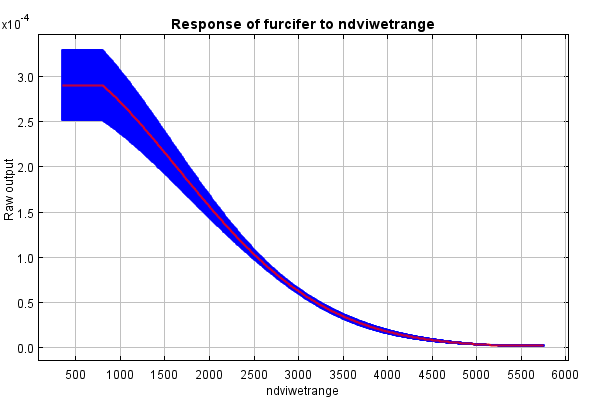 |
| Patch Size  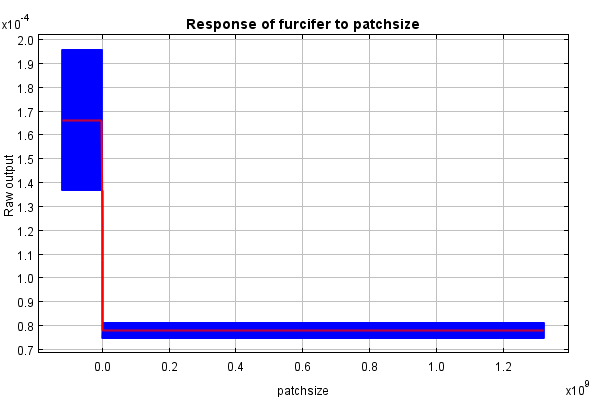 |  |

***Ae.luteocephalus,* November-Only 2010 Model**

| Distance From Patch Edge  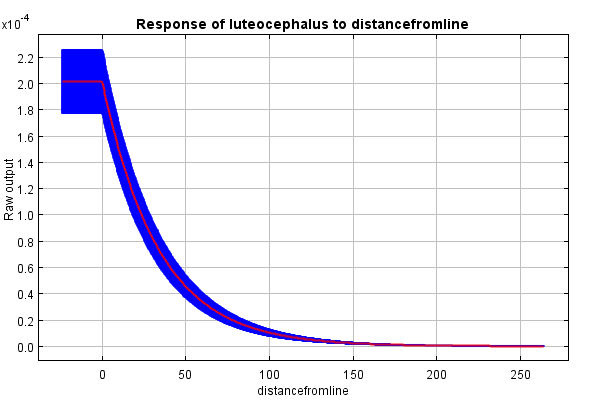 | Distance from Large Forest Patches  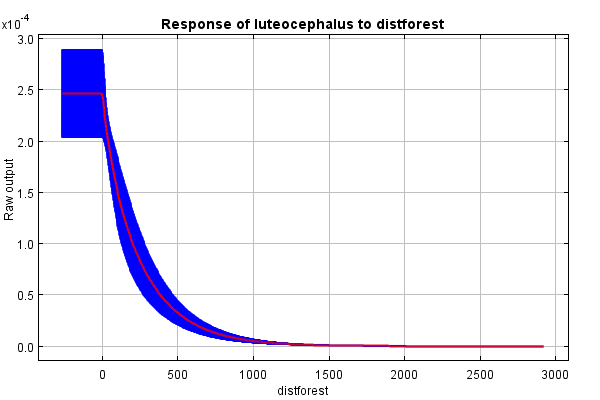 |
| --- | --- |
| Patch Size  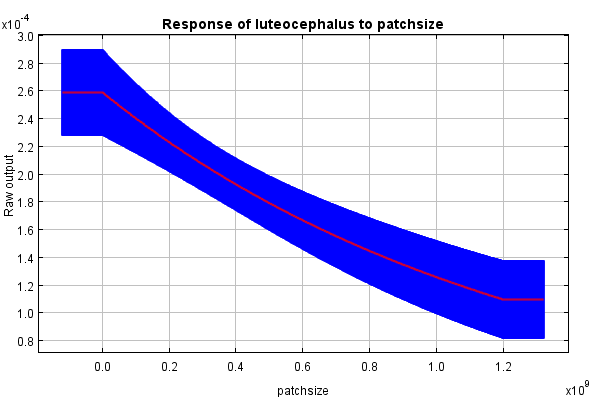 | Precipitation of the Wettest Quarter  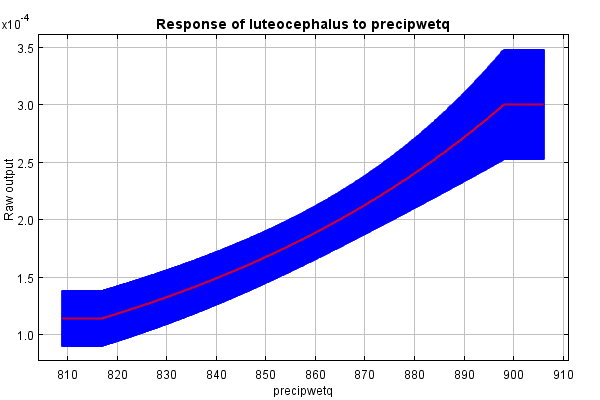 |

***Ae.taylori,* November-Only 2010 Model**

| Mean NDVI for 2009 and 2010 October-November  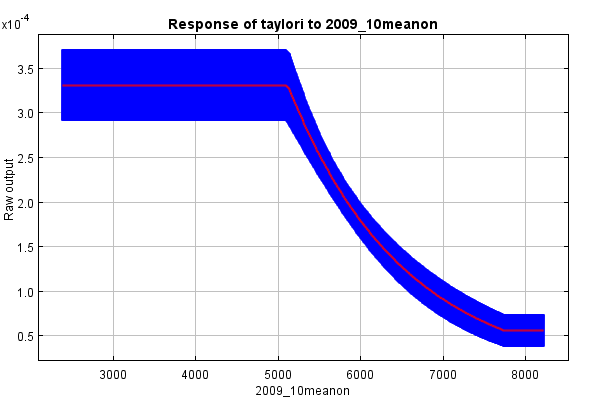 | Distance from Large Forest Patches  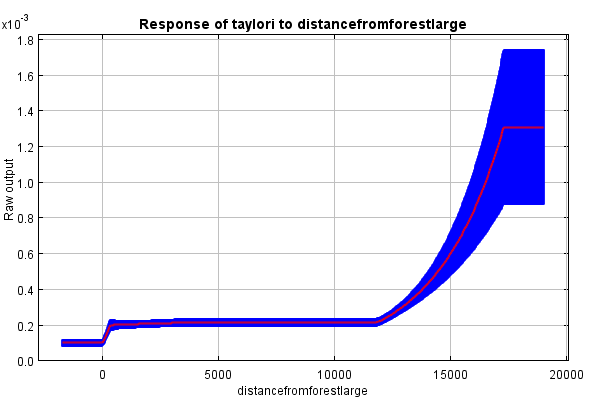 |
| --- | --- |
| Distance From Patch Edge  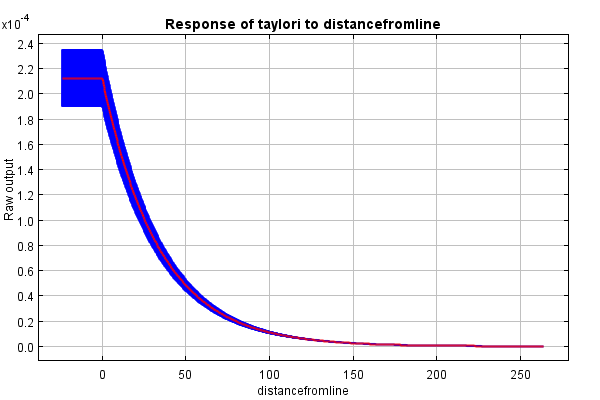 | Distance from Any Forest Patch  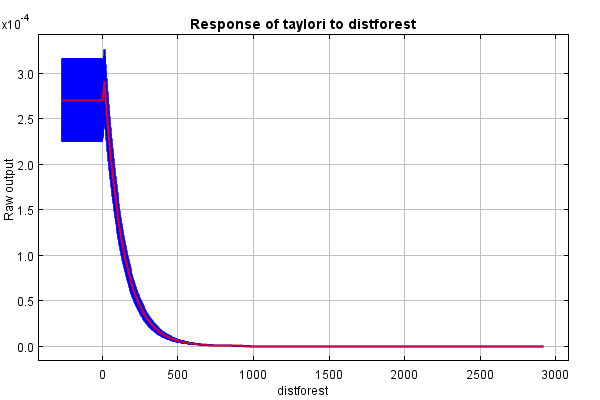 |
| Range of NDVI for 2009 and 2010 Rainy Seasons  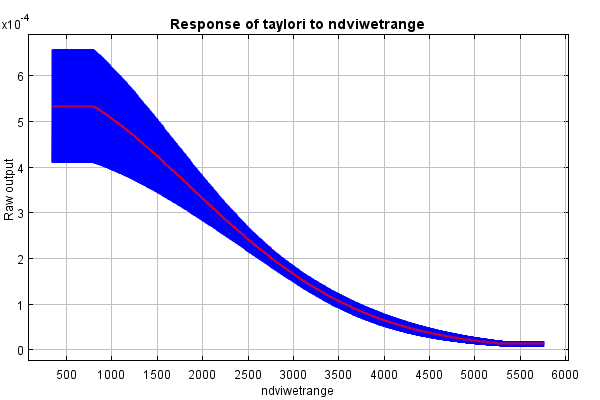 | Patch Size  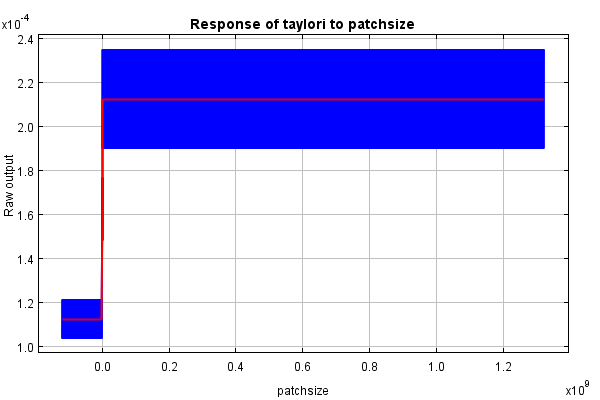 |
| Precipitation of the Wettest Quarter  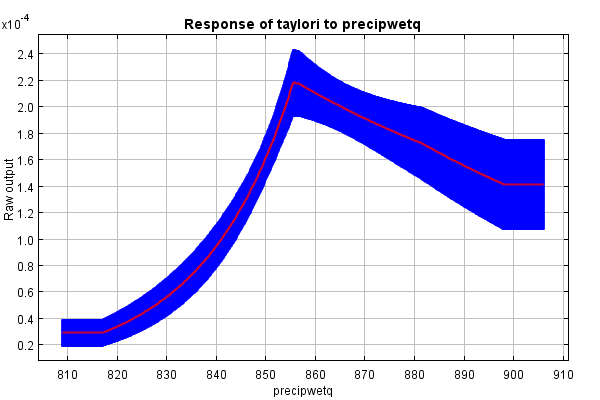 |  |

***Ae.vittatus,* November-Only 2010 Model**

| Mean NDVI for 2009 and 2010 October-November  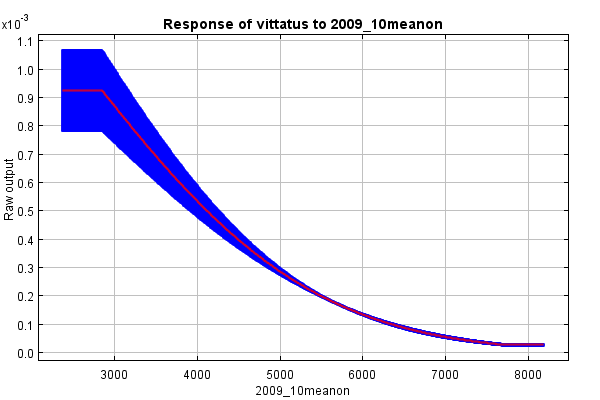 | Distance From Patch Edge  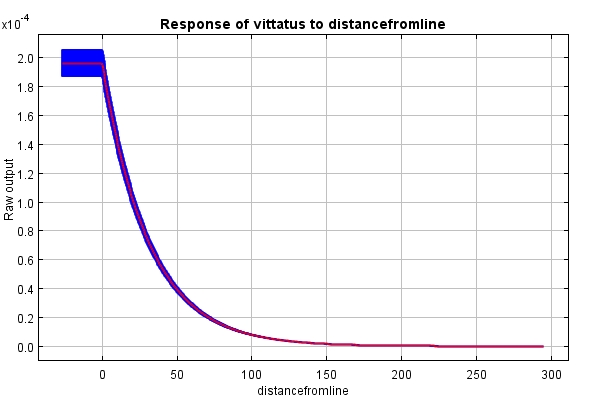 |
| --- | --- |
| Distance from Any Forest Patch  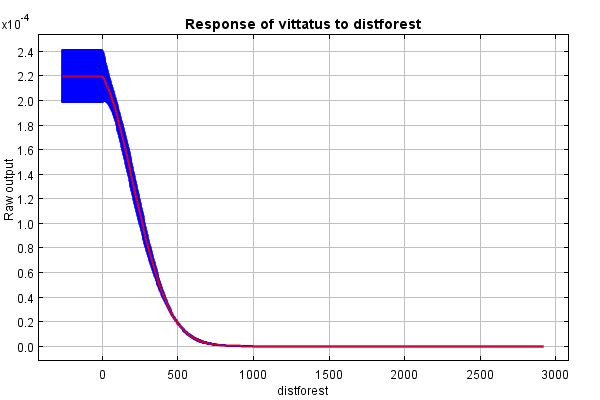 | Slope  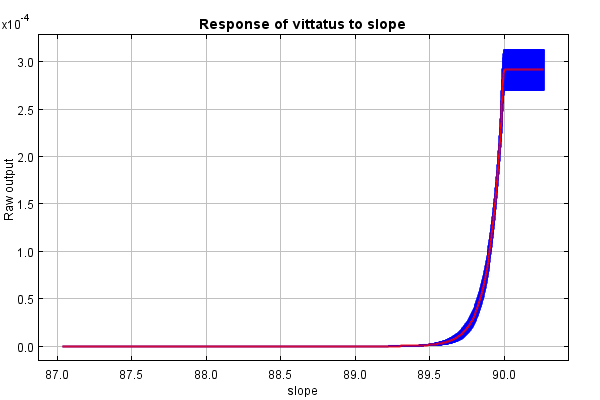 |
